# Coumarin Triazoles as Potential Antimicrobial Agents

**DOI:** 10.3390/antibiotics12010160

**Published:** 2023-01-12

**Authors:** Siddappa A. Patil, Aravind R. Nesaragi, Raúl R. Rodríguez-Berrios, Sydney M. Hampton, Alejandro Bugarin, Shivaputra A. Patil

**Affiliations:** 1 Centre for Nano and Material Sciences, Jain University, Jain Global Campus, Bangalore 562112, Karnataka, India; 2 Department of Chemistry, University of Puerto Rico, Rio Piedras Campus, P.O. Box 23346, San Juan 00931-3346, Puerto Rico; 3 Department of Chemistry and Physics, Florida Gulf Coast University, 10501 FGCU Boulevard South, Fort Myers, FL 33965, USA; 4 Pharmaceutical Sciences Department, College of Pharmacy, Rosalind Franklin University of Medicine and Science, 3333 Green Bay Road, North Chicago, IL 60064, USA

**Keywords:** carbazole, triazole, antimicrobial, antifungal, antibacterial, drug resistance

## Abstract

Currently, in hospitals and community health centers, microbial infections are highly common diseases and are a leading cause of death worldwide. Antibiotics are generally used to fight microbial infections; however, because of the abuse of antibiotics, microbes have become increasingly more resistant to most of them. Therefore, medicinal chemists are constantly searching for new or improved alternatives to combat microbial infections. Coumarin triazole derivatives displayed a variety of therapeutic applications, such as antimicrobial, antioxidant, and anticancer activities. This review summarizes the advances of coumarin triazole derivatives as potential antimicrobial agents covering articles published from 2006 to 2022.

## 1. Introduction

In the modern drug discovery era, the design and development of new antimicrobial drugs are receiving great attention from the research community due to the emergence of multidrug-resistant strains (MDRs) in recent years [1,2,3,4]. MDRs pose a serious health threat to the global population and are frequently associated with increased healthcare costs and prolonged hospital stays [5]. Even though recent advances have improved our understanding of the pathogenesis of antimicrobial infection, scientists have become increasingly focused on discovering novel, more effective, and safe drug Candidates to overcome MDRs. In recent years our research lab has been actively involved in the design and development of new bioactive molecules to tackle MDR strains [6,7,8,9,10,11,12,13,14].

**Figure 1:**
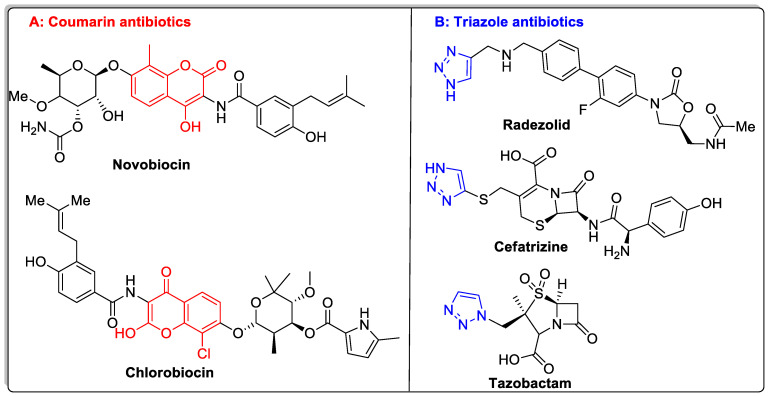
Clinically used important (**A**) coumarin and (**B**) triazole-based antibiotic drugs.

Coumarin pharmacophore has been considered the most ideal small-molecule scaffold for the development of new drugs because of its drug-like properties and, more significantly, its association with innumerable pharmacological activities. Coumarin pharmacophore is part of several clinically used drug Candidates, including some well-known antibiotic drugs (Figure 1A). Our lab recently comprehensively reviewed the medicinal applications of pharmacologically important coumarins [15,16].

**Figure 2:**
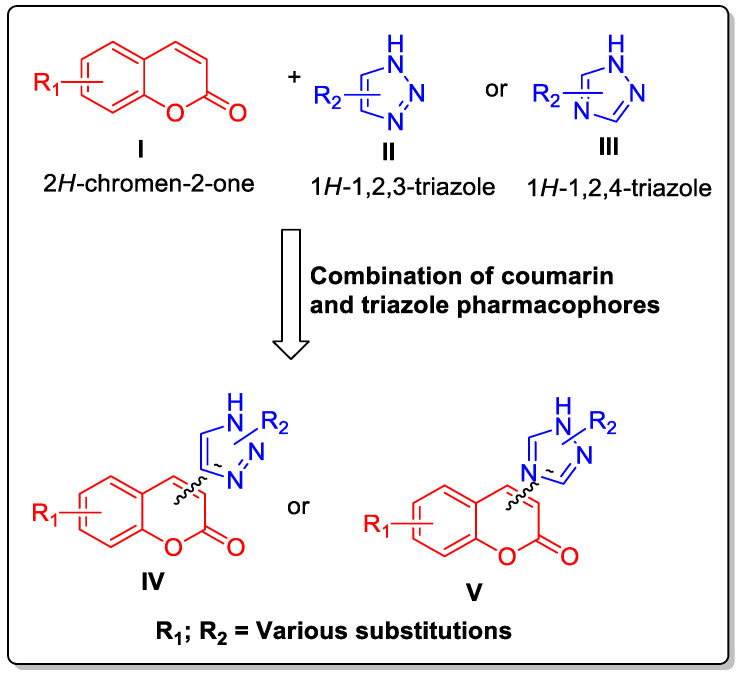
Combination of coumarin and triazole moieties to obtain a more effective single-drug molecule.

Triazole, also recognized as pyrrodiazole, is a five-membered nitrogen heterocycle with two carbon and three nitrogen atoms. Triazole exists in two isomeric forms—1,2,3-triazole (**II**) and 1,2,4-triazole (**III**)—based on the positions of the nitrogen atoms in the five-membered ring system (Figure 2). Triazole analogs have greatly attracted biologists and chemists alike due to their wide applications in medicinal chemistry with numerous biological activities [17,18,19,20]. Triazole moiety is part of several clinically used drugs for the treatment of various illnesses such as cancer, diabetes, etc. Some notable antimicrobial drugs have been listed in Figure 1B.

The combination of two or more clinical drugs to achieve higher efficacy and greater clinical benefits is becoming the new normal in clinical trials. Thus, combinatorial therapies are becoming a very important part of the clinical trial process to achieve success in patient well-being. Keeping this in mind, drug discovery researchers are planning to combine two or more drug functionalities in a single molecule to obtain synergistic effects or to enhance the particular pharmacological effects of drug Candidates. Considering the pharmacological importance of both coumarins and triazoles, medicinal chemists have worked to develop new small-molecule drugs by combining coumarin (**I**) and triazole moieties (**II** or **III**) to generate more effective drugs (**IV** and **V**) (Figure 2).

From the literature, we observed increased antimicrobial activities by the insertion of a triazole ring into the various organic core molecules. Most of the existing antimicrobial drugs hold triazole pharmacophore in their elemental structures, which proves the antimicrobial potencies of the triazole template so that it expresses significant antimicrobial activity. From the in silico studies, it is evident that the enzyme forms hydrogen bonding interactions with the triazole ring along with coumarin moiety. Since both lactone (coumarin) and triazole are bioactive pharmacophores, the new hybrid molecule with these two bioactive species will be with increased effects evaluated in comparison to the parent drug.

The present article covers the antimicrobial activities of the combined coumarin and triazole analogs published to date and serves to further advance the drug design and development process of coumarin-bearing triazoles as possible new drug Candidates to overcome the effects of the MDR strains.

## 2. Antibacterial and Antifungal Activities of Coumarin Triazole Derivatives

**Figure 3:**
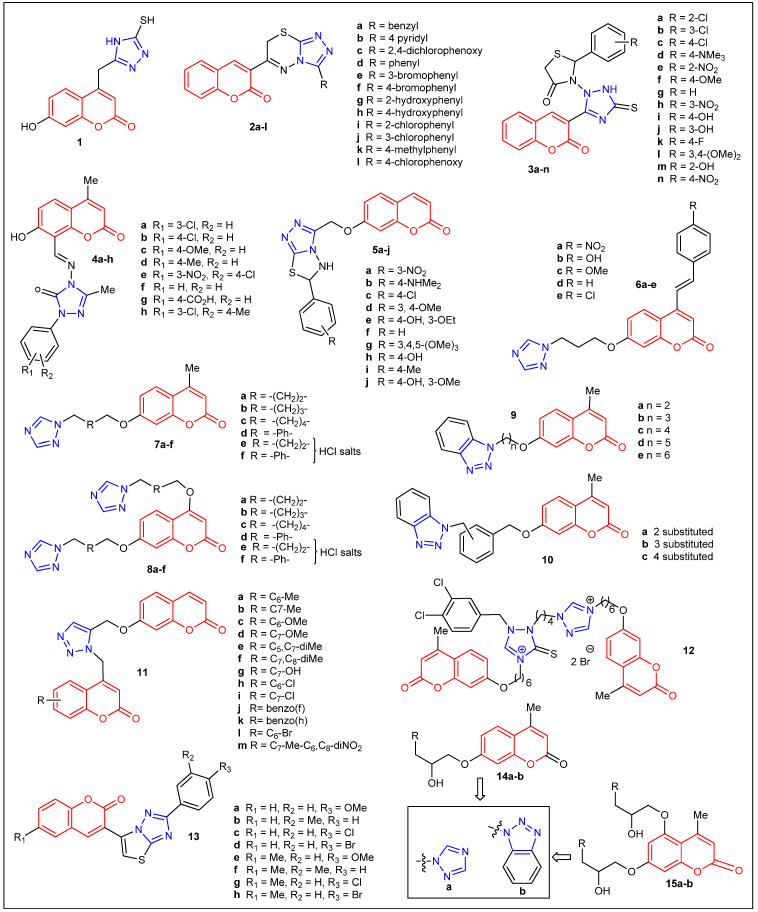
Structures of the reported coumarin triazole derivatives from **2006**–**2014**.

In 2006, M. Cacic et al. reported the first example of a C4-triazole-substituted coumarin **1** (Figure 3) together with its antibacterial activity [21]. Examination of the antimicrobial activity of **1** indicated high antimicrobial activity against *S. pneumoniae*, and it was slightly less active against *P. aeruginosa*, *B. subtilis*, *B. cereus*, and *S. panama*. The authors did not report the exact values of antimicrobial activity data and concluded their results with a generalized viewpoint. Furthermore, they noted that the research was in progress. A year later, Jayashree et al. reported the synthesis, characterization, and antimicrobial activity of twelve C-3-substituted triazolo-thiadiazinyl coumarin derivatives **2a**–**l** from salicylaldehyde as a starting material (Figure 3) (Table 1) [22]. The antibacterial screening demonstrated that compounds **2a**, **2b**, and **2c** had a comparable activity with the standard antibiotics (amoxicillin and gentamycin) against two species of Gram-positive bacteria (*B. subtilis* and *S. aureus*) and three species of Gram-negative bacteria (*E. coli*, *K. pneumoniae*, and *P. aeruginosa*). Overall, aryl substitution has improved the antimicrobial activity compared to their corresponding heteroaryl analogs. Compound **2a** displayed a 38 mm zone of inhibition (ZoI) toward *B. subtilis*, 35 mm (*K. pneumoniae*), and 32 mm (*S. aureus* and *E. coli).* Their most active compound, **2b**, exhibited the ZoI toward *S. aureus* (43 mm), *B. subtilis*, *K. pneumoniae*, *P. aeruginosa* (42 mm), and *E. coli* (40 mm). 

In 2009, the synthesis and characterization of fourteen C-3-substituted triazolothiazolidinone derivatives of coumarin **3a**–**n** were reported by Mashooq A. Bhat et al. (Figure 3) (Table 1) [23]. Compounds with Cl substitution, **3b** and **3c**, showed the highest activity against *S. aureus* (ZoI = ~20 mm). In addition, analogs with N(Me)_2_ (**3d**), NO_2_ (**3e**), OMe (**3f**), and Cl (**3a**, **3b**, and **3c**) substitutions displayed the highest activity against *C. albicans* (ZoI = ~18 mm). Interestingly, the compound without substitutions **3g** showed broad growth inhibition against *S. aureus*, *E. coli*, and *C. albicans.* Although all the adducts exhibited modest to good inhibition, none of them were superior to the standards ciprofloxacin (ZoI = 25 mm) or ketoconazole (ZoI = 20 mm). In addition, in 2019, Kotresh et al. reported the synthesis and antimicrobial properties of eight coumarin C-8-substituted Schiff Bases Triazole Derivatives (Figure 3) (Table 1) [24]. The highest antibacterial activity against *B. subtilis* and *E. coli* was obtained by compounds **4a** and **4b** (ZoI = ~18 mm), but less so than the reference drug norfloxacin (ZoI = 22 mm). Adducts **4a**, **4c**, **4d**, and **4e** showed good antifungal activity toward *A. niger* and *C. albicans* (ZoI = 18–22 mm) but lower than the standard griseofulvin (ZoI = 26 mm). Both electron-withdrawing groups (chloro, nitro) and electron-donating groups (methoxy, methyl) on the aryl ring might have contributed to the effectiveness of the particular strains. In general, the results indicated that the majority of the C-8-substituted coumarin compounds might serve as better fungicides than bactericides. In 2010, P. M. Kumar et al. employed microwave irradiation to synthesize ten coumarinyl-triazolothiadiazoles derivatives (**5a**–**j**) in high-yield and short-reaction times [25]. These compounds (Figure 3) (Table 1) were screened in vitro for their antibacterial and antioxidant activity. Particularly, compound **5a** (R = 3-nitrophenyl) showed the greatest antibacterial activity against *S. aureus* (10–15 mm inhibition diameter) and *E. coli* (16–22 mm inhibition diameter), while compounds **5b** (4-dimethylaminophenyl) and **5c** (4-chlorophenyl) showed moderate activity (10–15 mm inhibition diameter). Compounds **5a** (R = 3-nitrophenyl), **5d** (3,4-dimethoxyphenyl), and **5e** (4-hydroxy-3-ethoxyphenyl) displayed moderate antifungal activity toward *C. albicans* (10–15 mm inhibition diameter). Unfortunately, none of them showed superior activity when compared to the standard ciprofloxacin and fluconazole. G. R. Kokil et al. attached to 7-hydroxy-4-methylcoumarin a triazole moiety and a substituted aromatic ring at the C-7 and C-4 positions, respectively (Figure 3) (Table 1) [26]. The resulting 1,2,4-triazole coumarin derivatives were screened for their in vitro antifungal activity against *C. albicans ATCC 24433*. Compound **6a** (R = 4-NO_2_) showed good antifungal activity (MIC = 12.5 μg/mL), which was comparable with the standard drug ketoconazole (MIC = 12.5 μg/mL). The other compounds, such as **6b** (R = 4-OH) and **6c** (R = 4-OCH_3_), showed moderate antifungal activity.

In 2011, the synthesis and in vitro antimicrobial evaluation of two series of coumarin-mono- and bis-triazoles derivatives **7a**–**f** and **8a**–**f** were reported by Y. Shi and C. H. Zhou (Figure 3) (Table 1) [27]. Particularly, bis-triazole **8a** and its hydrochloride **8e** gave the most potent antimicrobial efficacy (MIC = 1–4 μg/mL) against four Gram-positive bacteria (*S. aureus* ATCC 25923, (MRSA), *B. subtilis* ATCC 6633, and *M. luteus* ATCC 4698), four Gram-negative bacteria (*E. coli* ATCC 25922, *P. vulgaris* ATCC 6896, *S. typhi* ATCC 9484 and *S. dysenteriae* ATCC 49550); as well as three fungi (*C. albicans* ATCC 76615, *S. cerevisiae* ATCC 9763, and *A. fumigatus* ATCC 96918). Other mono-triazole compounds **7a**–**c**, bis-triazole **8a**–**c**, hydrochloride mono-triazole **7e**–**f**, and hydrochloride bis-triazole **7e**–**f** showed comparable or superior anti-MRSA activity than the clinical antibacterial drugs enoxacin (MIC = 1–4 μg/mL) and chloromycin (MIC = 4–16 μg/mL). Compounds **7a**, **8a**, and **8e** exhibited comparable antifungal potency against *C. albicans* and *S. cerevisiae* (MIC = 2–4 μg/mL) than the positive control fluconazole (MIC = 1–2 μg/mL) and showed strongest inhibition toward *A. fumigatus* (MIC = 2–48 μg/mL), whereas fluconazole gave MIC = 128 μg/mL. In conclusion, the alkyl linker has provided better activity compared to the phenyl linker in both monomers as well as dimear triazolo-coumarins. In general, coumarin-bis-triazoles **7** exhibit stronger antimicrobial efficiency compared to their corresponding mono-triazole derivatives **8**. The authors pointed out that water-soluble hydrochloride salts have shown stronger antibacterial and antifungal efficacy in comparison with their corresponding poor water-soluble triazole precursors. They postulated that the conversion of triazoles into their hydrochlorides could modulate the lipid/water partition coefficient, affect their diffusion in bacterial cells, as well as interact with bacterial cells and tissues. Thus, water-soluble salts might improve the pharmacological properties of these new triazole analogs. They assume that further studies will help to understand the mechanism of actions of these derivatives.

Shi Yuan et al. also reported the synthesis of two series of coumarin-based benzotriazole derivatives (**9** and **10**) via a multi-step sequence (Figure 3) (Table 1) and studied the in vitro antimicrobial activities against four Gram-positive bacteria, four Gram-negative bacteria, and three fungi [28]. Compounds **9a**–**e** and **10a**–**c** were more active (MIC = 4–8 μg/mL) than chloromycin (MIC = 16 μg/mL) on *P. vulgaris* ATCC 6896. Coumarin benzotriazoles **9a (n = 2; CH_2_-CH_2_)** and **10b (3-substituted)** displayed comparable antibacterial efficacy against *S. aureus* ATCC 25923 and *M. luteus* ATCC 4698 in comparison with the reference drug chloromycin (MIC = 4 μg/mL). Compared to fluconazole (MIC = 128 μg/mL), compounds **10a**–**d** showed stronger inhibition against *A. fumigatus* ATCC 96918 (MIC = 64 μg/mL). More importantly, fluconazole-insensitive *A. fumigatus* and methicillin-resistant *S. aureus* N 315 (MRSA) were sensitive to the new adducts.

In 2012, Naik et al. employed click chemistry as a means to synthesize thirteen 1,4-disubstituted bis-chromenyl triazole coumarin derivatives **11a**–**m** and studied their antimicrobial activity (Figure 3) (Table 1) [29]. Only three compounds **11h**–**j** showed antitubercular activity against *M. tuberculosis*, equivalent to the activity of *streptomycin*, with a MIC value of 6.25 μg/mL. Compound **11c** (C6-Methoxy) showed higher antifungal activity (MIC = 6.25 μg/mL) than fluconazole (MIC = 8 μg/mL) against *A. niger*. In summary, all the compounds were better antitubercular agents than antimicrobial agents. However, they showed modest activity against Gram-positive bacteria [*S. faecalis* (MTCC 3382) and *S. aureus* (MTCC 3160)] and Gram-negative bacteria [*P. aeruginosa* (MTCC 1034) and *E. coli* (MTCC 1089)]. The synthesis of thio-triazole derivative **12** (Figure 3) and its in vitro antibacterial and antifungal activities were reported by Wang and coworkers [30]. This coumarin thio-triazole salt showed good antimicrobial activities (MIC = 8–32 μg/mL) against MRSA (N315), *S. aureus* (ATCC25923), *B. subtilis* and *M. luteus* (ATCC4698), *E. coli* (DH52), *E. typhosa*, and *C. albicans* (ATCC76615) and low efficiency (MIC = 128 μg/mL) toward *S. dysenteriae*, *P. aeruginosa*, and *C. mycoderma*. A green synthesis of 2-aryl-5-(coumarin-3-yl)-thiazolo [3,2-*b*][1,2,4]-triazoles **13a**–**h** (Figure 3), using microwave irradiations under solvent-free conditions, was reported by K. Jakhar and J. K. Makrandi (Table 1) [31]. All compounds displayed low to good inhibition (ZoI = 9–16 mm) against Gram-negative bacteria; *E. coli*, *P. aeruginosa*, *K. pneumoniae*, and *S. typhi*. Only compounds **13a**, **13b**, **13c**, and **13h** exhibited activity against the tested Gram-positive bacteria *S. aureus* (ZoI = 9–12 mm). It seems as if both methoxy and halogen substitution on the phenyl ring with methyl substitution on the coumarin ring showed the best activity. 

**Table 1:** Antimicrobial activity data of reported coumarin triazole derivatives.

Compound	Activity Observed	Bacteria/Fungal	Ref.	Compound	Activity Observed	Bacteria/Fungal	Ref.
**2a**	9 (nm)	*B. subtilis* and *S. aureus*	[22]	**6a**	200 (µg/mL)	*C. albicans*	[26]
**2b**	35 (nm)	*K. pneumoniae*	[22]	**6b**	25 (µg/mL)	*C. albicans*	[26]
**2c**	12 (nm)	*B. subtilis* and *E. coli*	[22]	**6c**	12.5 (µg/mL)	*C. albicans*	[26]
**2d**	19 (nm)	*B. subtilis*	[22]	**6d**	75 (µg/mL)	*C. albicans*	[26]
**2g**	8 (nm)	*S. aureus*	[22]	**6e**	37.5 (µg/mL)	*C. albicans*	[26]
**2h**	16 (nm)	*B. subtilis* and *E. coli*	[22]	**Ketoconazole**	12.5 (µg/mL)	*C. albicans*	[26]
**2i**	10 (nm)	*B. subtilis*	[22]	**7a**	16 (µg/mL)	*P. vulgaris*, *S. typhi*, *S. dysenteriae*, and *A. fumigatus*	[27]
**2j**	43 (nm)	*S. aureus*	[22]	**7b**	32 (µg/mL)	*E. coli*, *P. vulgaris*, and *S. dysenteriae*	[27]
**2k**	26 (nm)	*B. subtilis*	[22]	**7c**	32 (µg/mL)	*E. coli*, *P. vulgaris*, *S. typhi*, *S. dysenteriae*, *S. cerevisiae*, and *A. fumigatus*	[27]
**2l**	34 (nm)	*P. aeruginosa*	[22]	**7d**	64 (µg/mL)	MRSA, *E. coli*, *P. vulgaris*, *S. typhi*, *S. dysenteriae*, *S. cerevisiae typhi S*, and *A. fumigatus*	[27]
**Amoxicillin**	40 (nm)	*P. aeruginosa*	[22]	**7e**	64 (µg/mL)	*S. dysenteriae*	[27]
**Gentamycin**	41 (nm)	*P. aeruginosa*	[22]	**7f**	64 (µg/mL)	*E. coli*, *P. vulgaris*, *S. typhi*, *S. dysenteriae*, *S. cerevisiae*, and *A. fumigatus*	[27]
**3a**	16 (nm)	*C. albicans*	[23]	**8a**	4 (µg/mL)	*A. fumigatus*	[27]
**3b**	18 (nm)	*C. albicans*	[23]	**8b**	32 (µg/mL)	*A. fumigatus*	[27]
**3c**	16 (nm)	*C. albicans*	[23]	**8c**	32 (µg/mL)	*A. fumigatus*	[27]
**3d**	14 (nm)	*C. albicans*	[23]	**8d**	64 (µg/mL)	MRSA *B. subtilis*, *M. luteus*, *E. coli*, *S. dysenteriae*, and *A. fumigatus*	[27]
**3e**	17 (nm)	*C. albicans*	[23]	**8e**	2 (µg/mL)	MRSA, *P. vulgaris*, *S. cerevisiae*, and *A. fumigatus*	[27]
**3f**	16 (nm)	*S. aureus*	[23]	**8f**	16 (µg/mL)	MRSA *B. subtilis*, *M. luteus*, *P. vulgaris*, *S. typhi*, *S. dysenteriae*, and *S. cerevisiae A. fumigatus*	[27]
**3g**	16 (nm)	*E. coli*	[23]	**Enoxacin**	4 (µg/mL)	MRSA	[27]
**3h**	14 (nm)	*S. aureus* and *C. albicans*	[23]	**Chloromycin**	16 (µg/mL)	MRSA	[27]
**3i**	17 (nm)	*E. coli* and *C. albicans*	[23]	**Fluconazole**	128 (µg/mL)	*A. fumigatus*	[27]
**3j**	18 (nm)	*S. aureus*	[23]	**11a**	>100 (µg/mL)	S. faecalis, *P. aeruginosa*, and *E. coli*,	[29]
**3j**	18 (nm)	*C. albicans*	[23]	**11b**	>100 (µg/mL)	*P. aeruginosa* and *E. coli*,	[29]
**3k**	19 (nm)	*S. aureus*	[23]	**11c**	50 (µg/mL)	*S. faureus*, *S. aureus*, and *C. albicans*	[29]
**3l**	20 (nm)	*S. aureus*	[23]	**11d**	>100 (µg/mL)	*P. aeruginosa*	[29]
**3m**	23 (nm)	*S. aureus*	[23]	**11e**	>100 (µg/mL)	*P. aeruginosa*	[29]
**3n**	17 (nm)	*S. aureus*, *E. coli*	[23]	**11f**	>100 (µg/mL)	*P. aeruginosa*	[29]
**Ciprofloxacin**	25 (nm)	*S. aureus*	[23]	**11g**	>100 (µg/mL)	*P. aeruginosa*	[28]
**Ciprofloxacin**	25 (nm)	*E. coli*	[23]	**11h**	50 (µg/mL)	*S. faureus*, *E. coli*, and *C. albicans*	[29]
**4a**	17 (nm)	*A. niger*	[24]	**11i**	50 (µg/mL)	*S. faureus*, *P. aeruginosa*, *E. coli*, *C. albicans*, and *A. niger*	[29]
**4b**	23 (nm)	*C. albicans*	[24]	**11j**	50 (µg/mL)	*S. faureus*, *P. aeruginosa*, *E. coli*, *C. albicans*, and *A. niger*	[29]
**4c**	18 (nm)	*C. albicans*	[24]	**11k**	>100 (µg/mL)	*P. aeruginosa*,	[29]
**4d**	22 (nm)	*A. niger*	[24]	**11l**	>100 (µg/mL)	*P. aeruginosa*,	[29]
**4e**	18 (nm)	*A. niger*	[24]	**11m**	>100 (µg/mL)	*P. aeruginosa*,	[29]
**4f**	18 (nm)	*A. niger*	[24]	**Ciprofloxacin**	1 (µg/mL)	*S. faureus*, *S. aureus*, *P. aeruginosa*, and *E. coli*	[29]
**4g**	18 (nm)	*C. albicans*	[24]	**Fluconazole**	16 (µg/mL)	*C. albicans*	[29]
**4h**	21 (nm)	*C. albicans*	[24]	**12**	128 (µg/mL)	*S. dysenteriae*, *P. aeruginosa*, and *C. mycoderma*	[30]
**Norfloxacin**	22 (nm)	*E. coli*	[24]	**Chloromycin**	16 (µg/mL)	*P. aeruginosa*	[30]
**Norfloxacin**	22 (nm)	*B. subtilis*	[24]	**Norfloxacin**	4 (µg/mL)	MRSA and *E. typhosa*	[30]
**Griseofulvin**	26 (nm)	*A. niger*	[24]	**Fluconazole**	4 (µg/mL)	*C. mycoderma*	[30]
**Griseofulvin**	26 (nm)	*C. albicans*	[24]	**14a**	4 (µg/mL)	*C. utilis*, *C. albicans*, and *P. aeruginosa*	[32]
**5b**	16 (nm)	*E. coli*	[25]	**14b**	4 (µg/mL)	*C. albicans*	[32]
**5c**	10 (nm)	*E. coli*	[25]	**15a**	1 (µg/mL)	*C. albicans* and *E. coli*	[32]
**5d**	7 (nm)	*E. coli*	[25]	**15b**	8 (µg/mL)	*C. albicans*	[32]
**5e**	7 (nm)	*S. aureus* and *E. coli*	[25]	**Fluconazole**	1 (µg/mL)	*C. albicans*	[32]
**5h**	10 (nm)	*C. albicans*	[25]	**Chloromycin**	8 (µg/mL)	*M. luteus*	[32]
**5j**	10 (nm)	*C. albicans*	[25]	**Norfloxacin**	1 (µg/mL)	*P. aeruginosa*	[32]

In 2014, two series of coumarin triazoles **14a**,**b** and **15a**,**b** (Figure 3) were prepared and characterized by IR, NMR, MS, and HRMS spectra, and their in vitro biological activity with six bacteria and five fungi was evaluated (Table 1) [32]. Bis-triazole coumarin derivative **15a** showed the same anti-*C. utilis* activity (MIC = 4 μg/mL) to mono-triazole derivative **14a**, which makes those two adducts more potent than Fluconazole (MIC = 8 μg/mL). In addition, compound **14a** exhibited better activity against MRSA (MIC = 8 μg/mL) than **14b** (MIC = 32 μg/mL) and norfloxacin (MIC = 16 μg/mL). Compound **14b** showed very good activity (MIC = 4 μg/mL) toward *C. albicans*, and modest MIC values (16 μg/mL) were obtained for *C. utilis*, *C. mycoderma*, MRSA N315, *B. subtilis*, and *E. coli* JM109. Finally, **15b** showed lower or comparable antimicrobial activities than **14b** and the reference drugs mentioned above. Overall, mono-triazole substitution favors antimicrobial activity compared to bis-triazole coumarin analogs.

**Figure 4:**
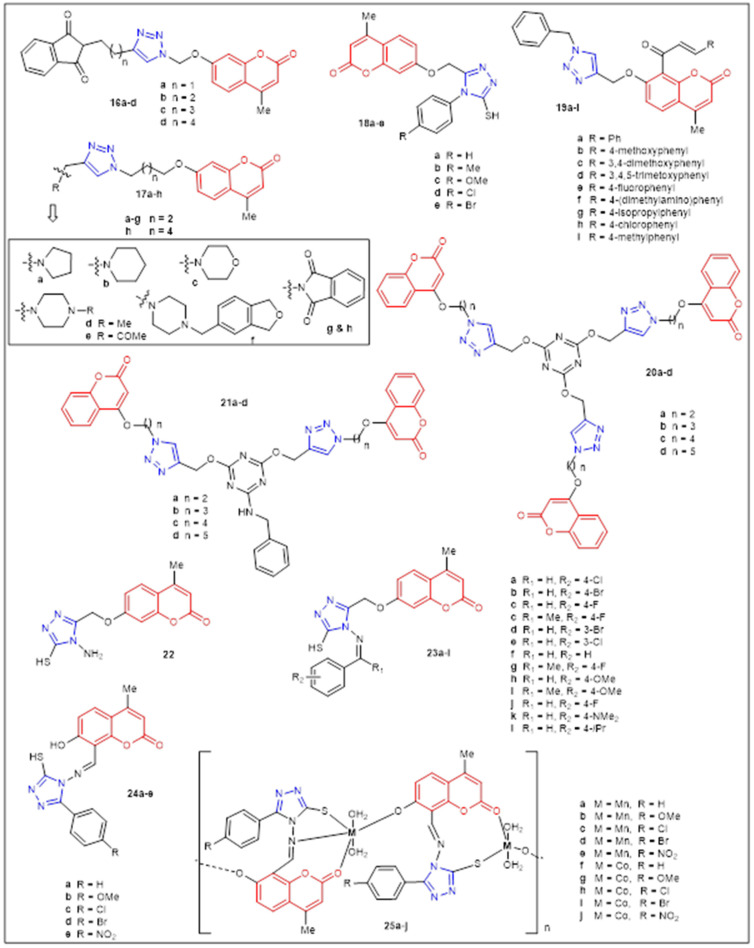
Structures of the reported coumarin triazole derivatives from **2014**–**2015**.

K. Kushwaha et al. reported the design and synthesis of coumarin-1,2,3-triazole derivatives **16a**–**d** and **17a**–**h** to study their antimicrobial properties (Figure 4) (Table 2) [33]. The majority of the compounds displayed similar antifungal activity toward *A. fumigatus* MTCC 343, *A. flavus* MTCC 277, and *C. albicans* MTCC 227 (ZoI = 12–23 mm). Remarkably, **16d** was the most active (ZoI = 23 mm) against *A. fumigatus*, and **16a** (n = 1; CH2) was the most active against *C. albicans* (ZoI = 20 mm), which was slightly better than the reference miconazole (ZoI = 15–19 mm). All the derivatives presented modest to good antibacterial activity against all the seven tested bacteria, albeit lower (ZoI = 10.5–15.7 nm) than the standard drug ciprofloxacin (ZoI = 18–20 nm). In general, compounds **17a**–**e** were selected as the best Candidates for further investigations due to their lower toxicity, high drug score values, and good oral bioavailability. Furthermore, in 2014, a group of C-7-triazole-substituted coumarins **18a**–**e** were synthesized with good yields and short reaction times using both microwave irradiation and grinding techniques (Figure 4) (Table 2) [34]. Compounds **18c**–**e** showed good antibacterial activity against *K. pneumonia* (ZoI = 16 mm), whereas adducts **18a**–**e** displayed moderate to good antimicrobial activity against *E. coli*, *A. niger*, *A. fumigates*, and *A. terrus* (ZoI = 6–12 mm).

Dongamanti et al. also reported a microwave-assisted synthesis of a series of hybrid compounds containing coumarin, 1,2,3-triazole, and chalcone substructures **19a**–**i** (Figure 4) which were screened for antimicrobial activity (Table 2) [35]. Derivatives **19c** and **19d** exhibit excellent activities against Gram-positive bacteria (*S. aureus* and *B. subtilis*) (ZoI = 32–35 mm) and Gram-negative bacteria (*E. coli* and *P. aeruginosa*) (ZoI = 31–33 mm) that are superior to the activities of the reference antibiotic amoxicillin (ZoI = 10–30 mm). Compounds **19b**, **19e**, and **19h** displayed good antibacterial activity, products **19f** and **19g** were moderately active, and derivatives **19a** and **19i** were weakly active in the antibacterial assay (ZoI = 4–17 mm). In regard to antifungal activity, adducts **19c**–**d** were more bioactive against *A. niger*, *F. oxysporum*, and *P. italicum* (ZoI = 13–30 mm) than the reference drug mycostatin (ZoI = 12–25 mm), while the other compounds were good to moderately active. In conclusion, dimethoxy and trimethoxy substitution yielded the highest activity toward several strains. Joshi et al. reported the synthesis and characterization of two series (**20a**–**d** and **21a**–**d**) of *s*-triazine-1,2,3-triazoles-coumarin dendrimers using click-chemistry (Figure 4) [36]. Compounds tris-(coumarin-1,2,3-triazole)*s*-triazines **20a**–**d** and bis-(coumarin-1,2,3-triazole)*s*-triazin-anilines **21a**–**d** were screened for antimicrobial activity against Gram-positive bacteria [*S. aureus* (MTCC96), *S. epidermidis* (MTCC435)], Gram-negative bacteria [*E. coli* DH5a and *P. aeruginosa* (MTCC434)] and fungal strains [*G. candidum*, *C. galbrata*, and *C. albicans*]. Adduct **20a** exhibited modest antifungal activities (% killing of 83) at a high concentration (250 μM) but displayed modest activity against all bacterial strains tested (values not shown) (Table 2). 

In 2015, A. M. Hayallah et al. documented the synthesis and antimicrobial activity of coumarin triazoles **22** and **23a**–**l** (Figure 4) (Table 2) [37]. The in vitro antibacterial activity was determined using *S. aureus* (AUMC B71) and *E. coli* (AUMC B69). In general, most of the newly-synthesized compounds exhibited moderate to good antibacterial activities compared to that of ciprofloxacin (20–30 vs. 40 ZoI). Specifically, compounds **22**, **23d**, and **23h** exhibited the same antibacterial activity against *E. coli* (MIC = 12.5 μmol/mL); however, **23d** was the most active against *S. aureus* (MIC = 25 μmol/mL), but lower than ciprofloxacin (MIC = 1.75 μmol/mL). Derivatives **22** and **23a**, **23e**, and **23f**–**j** were tested against *C. albicans* using fluconazole as a reference drug (MIC = 1.85 μmol/mL) and showed poor to null activity. Only compounds **23b**, **23c**, and **23d** showed antifungal activities (MIC = 25–50 μmol/mL). Kalwania et al. reported the synthesis, characterization, and antimicrobial activities of a 1,2,4-triazole-coumarin Schiff Bases **24a**–**e** and their Mn (II) and Co (II) complexes **25a**–**j** (Figure 4) (Table 2) [38]. Compounds **24a**–**e** and metal complexes **25a**–**j** were evaluated in vitro against five bacterial strains; *E. coli*, *P. aeruginosa*, *S. typhi*, *S. aureus*, and *B. subtilis*, using the standard drug gentamycin. Furthermore, the antifungal activities were evaluated against *A. niger* and *C. albicans* using fluconazole as the standard drug. All the Schiff bases **24a**–**e** demonstrated inferior antimicrobial activities with ZoI in the range of 45.21 mm to 78.32 mm toward all five bacterial and two fungal strains. However, their corresponding metal complexes **25a**–**j** showed higher antibacterial activity against selected bacteria, especially against *S. typhi* (ZoI: **25c**—79.36 mm; **25d**—76.44 mm; **25e**—82.05 mm; **25j**—80.00 mm). The metal complexes **25e** (ZoI: 76.09 mm and 79.23 mm) and **25j** (ZoI: 73.84 mm and 77.62 mm) have confirmed the antifungal activity toward *A. niger* and *C. albicans*, respectively. None of their compounds are comparable or superior to the standard drugs tested. In summary, metal complexes (**25**) have shown very good antimicrobial activity compared to their corresponding ligands (**24**).

**Table 2:** Antimicrobial activity data of reported coumarin triazole derivatives.

Compound	Activity Observed	Bacteria/Fungal	Ref.	Compound	Activity Observed	Bacteria/Fungal	Ref.
**16a**	20.2 (±1.69) mm	*C. albicans*	[33]	**Mycostatin**	20 mm	*P. italicum*	[35]
**16b**	21.3 (±1.90) (±) mm	*A. fumigatus*	[33]	**22**	30 mm	*E. coli*	[37]
**16c**	18.9 (±1.34) mm	*A. fumigatus*	[33]	**23a**	20 mm	*E. coli*	[37]
**16d**	23.4 (±1.97) mm	*A. fumigatus*	[33]	**23b**	20 mm	***Candida***	[37]
**17a**	18.5 (±0.70) mm	*A. fumigatus*	[33]	**23c**	19 mm	***Candida***	[37]
**17b**	18.8 (±1.13) mm	*A. fumigatus*	[33]	**23d**	30 mm	*E. coli*	[37]
**17c**	16.9 (±1.17) mm	*A. fumigatus*	[33]	**23e**	25 mm	*S. aureus*	[37]
**17d**	18.4 (±0.63) mm	*A. fumigatus*	[33]	**23f**	20 mm	*S. aureus*	[37]
**17e**	18.2 (±1.76) mm	*A. fumigatus*	[33]	**23g**	24 mm	*E. coli*	[37]
**17f**	20.6 (±0.91) mm	*A. fumigatus*	[33]	**23h**	28 mm	*E. coli*	[37]
**17g**	19.0 (±1.41) mm	*A. fumigatus*	[33]	**23i**	26 mm	*E. coli*	[37]
**17h**	18.5 (±0.70) mm	*A. fumigatus*	[33]	**23j**	20 mm	*E. coli*	[37]
**Ciprofloxacin**	20 mm	*S. epidermis*	[33]	**Ciprofloxcin**	40 mm	*S. aureus* and *E. coli*	[37]
**Miconazole**	19 mm	*C. albicans*		**Fluconazole**	40 mm	*Candida*	[37]
**18a**	12 mm	*K. pneumonia*	[34]	**24a**	64.73 mm	*S. typhi*	[38]
**18b**	12 mm	*K. pneumonia* and *Aspergillus terrs*	[34]	**24b**	70.31 mm	*C. albicans*	[38]
**18c**	16 mm	*K. pneumonia*	[34]	**24c**	76.44 mm	*S. typhi*	[38]
**18d**	16 mm	*K. pneumonia*	[34]	**24d**	72.96 mm	*S. typhi*	[38]
**18e**	16 mm	*K. pneumonia*	[34]	**24e**	78.32 mm	*S. typhi*	[38]
**Gentamycin**	18 mm	*K. pneumonia*	[34]	**25a**	68.13 mm	*S. typhi*	[38]
**Fluconazole**	13 mm	*A. niger* and *Aspergillus terrs*	[34]	**25b**	72.00 mm	*S. typhi*	[38]
**19a**	17 mm	*F. oxysporum*	[35]	**25c**	79.36 mm	*S. typhi*	[38]
**19b**	28 mm	*S. aureus* and *E. coli*	[35]	**25d**	76.44 mm	*S. typhi*	[38]
**19c**	32 mm	*S. aureus*	[35]	**25e**	82.05 mm	*S. typhi*	[38]
**19d**	35 mm	*S. aureus*	[35]	**25f**	65.00 mm	*S. typhi*	[38]
**19e**	27 mm	*S. aureus* and *E. coli*	[35]	**25g**	71.32 mm	*C. albicans*	[38]
**19f**	22 mm	*S. aureus*	[35]	**25h**	75.66 mm	*S. typhi*	[38]
**19g**	22 mm	*E. coli*	[35]	**25i**	72.22 mm	*S. typhi*	[38]
**19h**	25 mm	*S. aureus*	[35]	**25j**	80.00 mm	*S. typhi*	[38]
**19i**	18 mm	*F. oxysporum*	[35]	**Gentamycine**	100 mm	*E. coli*, *P. aeruginosa*, *S. typhi*, *S. aureus*, and *B. subtilis*	[38]
**Amoxicillin**	30 mm	*S. aureus* and *E. coli*	[35]	**Fluconazole**	100 mm	*A. niger* and *C. albicans*	[38]

**Figure 5:**
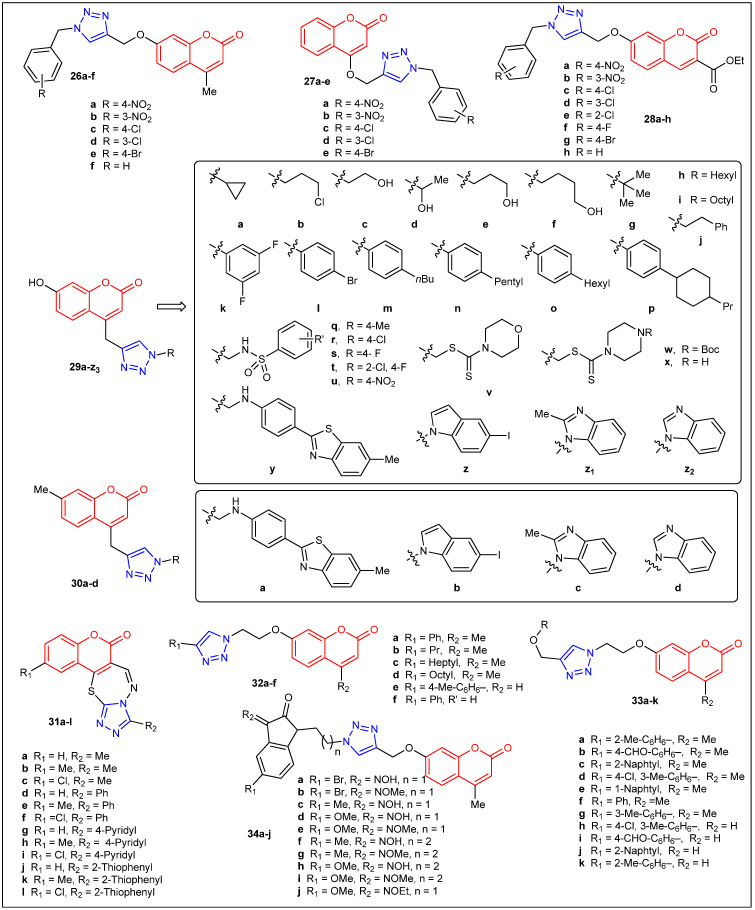
Structures of the reported coumarin triazole derivatives from **2016**–**2017**.

In 2016, Shingate et al. reported the synthesis of two sets of coumarin triazole derivatives [**26a**–**f** (7-subsituted) and **27a**–**e** (4-subsituted)], Figure 5 (Table 3). These new compounds were subjected to in vitro antimicrobial activity against three Gram-positive bacteria (*S. aureus*, *M. luteus*, and *B. cereus*), three Gram-negative bacteria (*E. coli*, *P. fluorescens*, and *F. devorans*), and three fungal strains (*A. niger*, *P. chrysogenum*, and *C. lunata*) [39]. All compounds showed modest to good antibacterial activity, but adduct **27a** was the most bioactive, with MIC values of 2 μg/mL against the three tested Gram-negative bacteria. Those were the same MIC values (2 μg/mL) obtained from the three standards used (ampicillin, kanamycin, and chloramphenicol). Similar results were observed from the antifungal study. However, this time adduct **26d** was the most superior compound among all, with MIC values of 4–8 μg/mL, which are comparable to those of the standards [miconazole (16 μg/mL), amphotericin B (2–16 μg/mL), and Fluconazole (2–4 μg/mL)]. The same year, Shingate et al. described the synthesis and antifungal activity of eight coumarin triazole derivatives [**28a**–**h** (7-substituted)], Figure 5 (Table 3) [40]. This time, the following five fungal stains were evaluated: *C. albicans*, *F. oxysporum*, *A. flavus*, *A. niger*, and *C. neoformans.* Compound **28c**, **28d**, **28e** (chloro-substituted), and **28h** were as potent as the standard drug miconazole against *C. albicans* (MIC = 25 μg/mL), while adduct **28f** showed twofold bioactivity when compared with miconazole and equally potent to fluconazole (MIC = 12.5 μg/mL). In order to identify the mechanism of action of these compounds, authors performed molecular docking studies with the active site of fungal *C. albicans* enzyme P450 cytochrome lanosterol 14α-demethylase. The highly effective compound **28f** exhibited the lowest interaction energy (−72.29 kcal/mol), and the standard drugs fluconazole and miconazole also showed good interaction energy that is −69.76 and −71.90 kcal/mol, respectively. Similarly, Raić-Malić et al. reported a straightforward synthesis (using click-chemistry to form the 1,2,3-triazole moiety) that produced 31 new coumarin triazole derivatives [**29a-z_2_** (4-substituted, 7-hydroxycoumarins) and **30a**–**d** (4-substituted, 7-methylcoumarins)], Figure 5 (Table 3) [41]. The relatively large library of compounds was screened against three Gram-positive bacteria [*S. aureus* (ATCC 25923), *E. faecalis*, vancomycin-resistant *E. faecium* (VRE)], and four Gram-negative bacteria [*P. aeruginosa* (ATCC 27853), *E. coli* (ATCC 25925), *A. baumannii* (ATCC 19606), and extended-spectrum β-lactamase (ESBL)-producing *K. pneumoniae*]. Unfortunately, none of the 31 adducts exhibited any bioactivity against the Gram-negative bacteria tested. In addition, among the 31 tested compounds, only 13 showed activity against two of the three Gram-positive bacteria examined [*E. faecalis* and vancomycin-resistant *E. faecium* (VRE)]. Nonetheless, coumarin 1,2,3-triazole hybrids **29n** (*p*-pentylphenyl), **29t** (2-chloro-4-fluorobenzenesulfonamide), and **29x** (dithiocarbamate) showed selective anti-Enterococcus species activities. For instance, those three compounds displayed MIC values of 64 μg/mL against vancomycin-resistant *E. faecium*, whereas the reference antibiotics ceftazidime and ciprofloxacin didn’t exhibit bioactivity (MICs were >256 mg/mL). Furthermore, adduct **29n** demonstrated superior inhibitory against *E. faecium* (MIC value of 8 μg/mL). Among this large pool of compounds, aryl and heteroaryl substitution on triazole moiety demonstrated greater activity, implying that the substitution on triazole is vital for obtaining better antimicrobial activity.

In 2017, the synthesis of twelve 1,2,4-triazolo-1,3,4-thiadiazepino-fused coumarins, together with their antimicrobial activity, was presented by Patel and co-workers [42]. To produce those 12 adducts (**31a**–**l**), Figure 5, the authors simply reacted three 4-chloro-3-formylcoumarins with four 4-amino-5-substituted-3-mercapto-1,2,4-triazoles in the presence of a base. All the adducts (**31a**–**l**) were evaluated against two Gram-positive bacteria, *S. aureus* (MTCC 96) and *B. subtilis* (MTCC 441), two Gram-negative bacteria, *E. coli* (MTCC 443) and *S. typhi* (MTCC 98), and two fungal strains, *C. albicans* (MTCC 227) and *A. niger* (MTCC 282) (Table 3). All compounds were inactive against all fungal strains (griseofulvin and nystatin were used as standard antifungal drugs). Only a few adducts (**31a**, **31e**, **31j**, and **31k**) showed antibacterial activity comparable to the standard drug ampicillin (MIC values around 100 μg/mL) but lower activity compared with chloramphenicol (MIC = 50 μg/mL) and Norfloxacin (MIC = 10 μg/mL) (Table 3). The adducts **31a, 31e,** and **31j** exhibited the MIC 62.5 μg/mL toward *E. coli*, whereas **31k** showed the MIC 62.5 μg/mL against *S. aureus*. Only the methyl substitution on coumarin and (thio)phenyl substitution on triazole moiety have produced the desired antimicrobial activity comparable to the standards used. In the same year, Pal et al. prepared 17 new coumarin triazole derivatives (**32a**–**f** and **33a**–**k**), Figure 5 [43]. All synthesized adducts were evaluated against one Gram-positive bacteria, *S. aureus*, and three Gram-negative bacteria, *E. coli*, *P. aeruginosa*, and *K. pneumonia* (MTCC 441). Although all compounds showed some inhibition at 11–18 mm (ZoI at 0.5 mg/100 μL) for all the tested bacterial strains, these values were lower than the reference drug pefloxacin (28–36 mm). Jin et al. published the synthesis and antimicrobial evaluation of 10 different triazole-tethered isatin–coumarin hybrids (**34a**–**j**), Figure 5 (Table 4) [44]. The following four Gram-positive bacterial strains: methicillin-sensitive S. epidermidis, methicillin-resistant S. epidermidis, methicillin-sensitive *S. aureus*, and methicillin-resistant *S. aureus* (ATCC) and four Gram-negative bacterial strains: extended-spectrum beta-lactamases (ESBLs)-producing *E. coli* ESBLs (−), *E. coli* ESBLs (+), *K. pneumoniae* ESBLs (+), and *K. pneumoniae* ESBLs (−) were used to evaluate the newly synthesized adduct. All adducts displayed poor to modest activity across the board (MIC range 16 to >200 μg/mL), whereas the reference drug ciprofloxacin showed a MIC range of 0.015 to 64 μg/mL. It is worth noting that those bacterial strains are resistant and adduct **34e** (n = 1; R_1_ = OMe; R_2_ = NOMe) showed 16 μg/mL (MIC) against methicillin-resistant S. epidermidis, which had a higher inhibition when compared to ciprofloxacin (MIC = 64 μg/mL).

**Figure 6:**
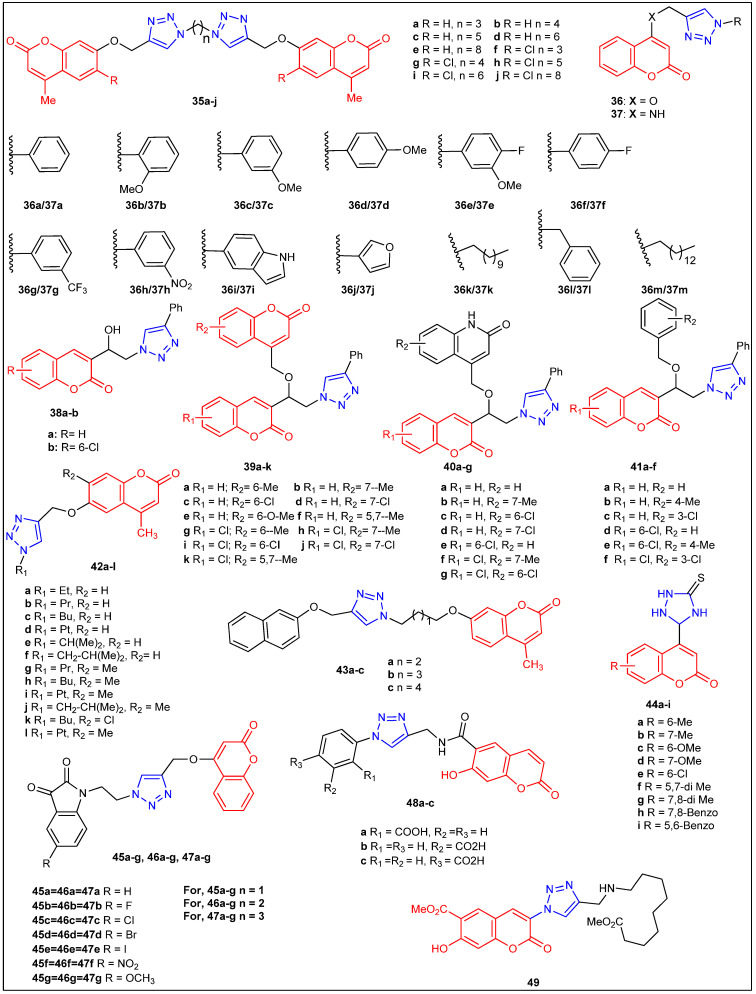
Structures of the reported coumarin triazole derivatives from **2018**–**2019**.

The microwave-aided synthesis of dimers of ten distinct coumarin-1,2,3-triazoles containing an alkyl spacer (**35a**–**j**) was reported by Ashok et al. in 2018 [45] (Figure 6). The synthesized compounds were screened for their antimicrobial activity against two Gram-positive strains, *B. subtilis* (ATCC 6633) and *S. aureus* (ATCC 6538), two Gram-negative strains, *E. coli* (ATCC 11229) and *P. vulgaris* (ATCC 29213), and two fungal strains, *A. niger* (ATCC 9029) and *C. albicans* (ATCC 10231). The compound **35j** showed MIC values of 3.125–6.25 µg/mL and 12.5 µg/mL, four bacterial and two fungal strains, respectively. The compound **35j** was discovered to be more effective than the other investigated compounds against the tested bacterial and fungal strains. Except for compound **35j**, compounds **35e** and **35i** demonstrated modest activity against bacterial strains with MIC values of 6.25–12.5 µg/mL. Compounds **35d**, **35e**, and **35i** displayed better antifungal activity with MIC values of 12.5–25 µg/mL. Coumarin–triazoles with alkyl linker (n = 6 and 8) have produced comparable antibacterial (**35e** and **35i**) as well as antifungal activity, indicating that the long linker could have played a role in getting the desired activity. López- Rojas et al. [46] reported a series of coumarin-1,2,3-triazole derivatives with diverse alkyl, phenyl, and heterocycles at C-4 of the triazole nucleus via copper(I)-catalyzed Huisgen 1,3-dipolar cycloaddition reaction (**36a**–**m** and **37a**–**m**) (Figure 6) (Table 4). The antibacterial activity of each molecule was evaluated against Gram-positive bacteria, *B. subtilis*, *S. aureus*, and *E. faecalis*, Gram-negative bacteria, *E. coli*, *P. vulgaris*, *K. pneumonia*, *P. aeruginosa*, and the fungus *C. albicans* for antifungal activity. Compounds **36a**, **36b**, **36f**, **37h,** and **37k** exhibited potential activity against *E. faecalis* at MICs ranging from 2.5 to 50.0 µg/mL. The most effective compound was found to be **36b,** with the 2-OMe-Ph group linked to the triazole nucleus and an OCH_2_ linker. In contrast, the comparable isoster **37b** (-NHCH_2_-) was found to be 64-fold less active than **36b**. Subsequently, compounds **36c** (3-OMe-Ph) and **36d** (4-OMe-Ph) had 8- and 16-fold less antibacterial activity than **36b,** respectively. The location of the OMe group on the phenyl ring also plays a significant influence on the activity. In order to be a successful antimicrobial drug Candidate, it should display the least toxicity toward normal cells. The authors evaluated the active compounds **36a**, **36b**, **36f**, **37h,** and **37k** for toxicity (hemolytic activity) against human erythrocytes, and all tested compounds demonstrated low toxicity toward human erythrocytes.

**Table 3:** Antimicrobial activity data of reported coumarin triazole derivatives.

Compound	Activity Observed	Bacteria/Fungal	Ref.	Compound	Activity Observed	Bacteria/Fungal	Ref.
**26a**	2 µg/mL	*E. coli* and *P. fluorescens*	[39]	**29t**	32 µg/mL	*E. faecalis*	[41]
**26b**	2 µg/mL	*P. fluorescens*	[39]	**29u**	256 µg/mL	*E. faecalis*	[41]
**26c**	2 µg/mL	*F. devorans*	[39]	**29v**	32 µg/mL	*E. faecalis*	[41]
**26d**	2 µg/mL	*F. devorans*	[39]	**29x**	16 µg/mL	*E. faecalis*	[41]
**26e**	4 µg/mL	*B. cereus*, *E. coli*, and *F. devorans*	[39]	**Ceftazidime**	0.5 µg/mL	*E. coli*	[41]
**26f**	4 µg/mL	*M. luteus*, *E. coli*, and *F. devorans*	[39]	**Ciprofloxacin**	<0.125 µg/mL	*P. aeurigonsa*, *E. coli*, and *A. baumanni*	[41]
**27a**	2 µg/mL	*E. coli*, *P. fluorescens*, and *F. devorans*	[39]	**31a**	62.5 µg/mL	*E. coli*	[42]
**27b**	4 µg/mL	*M. luteus*, *B. cereus*, *E. coli*, and *P. fluorescens*	[39]	**31b**	100 µg/mL	*S. aureus*	[42]
**27c**	4 µg/mL	*M. luteus*, *E. coli*, *F. devorans*, and *A. niger*	[39]	**31c**	100 µg/mL	*E. coli*	[42]
**27d**	4 µg/mL	*M. luteus*	[39]	**31d**	100 µg/mL	*E. coli*	[42]
**27e**	4 µg/mL	*M. luteus*, *E. coli*, and *F. devorans*	[39]	**31e**	62.5 µg/mL	*E. coli*	[42]
**Ampicillin**	2 µg/mL	*B. cereus*, and *P. fluorescens*	[39]	**31f**	125 µg/mL	*E. coli*	[42]
**Kanamycin**	2 µg/mL	*S. aureus*, *M. luteus*, *B. cereus*, *E. coli*, *P. fluorescens*, and *F. devorans*	[39]	**31g**	125 µg/mL	*B. subtilis* and *S. aureus*	[42]
**Chloramphenicol**	2 µg/mL	*S. aureus*, *M. luteus*, *B. cereus*, *E. coli*, *P. fluorescens*, and *F. devorans*	[39]	**31h**	250 µg/mL	*B. subtilis*, *S. aureus*, and *E. coli*	[42]
**Miconazole**	16 µg/mL	*A. niger*, *P. chrysogenum*, and *C. lunata*	[39]	**31i**	250 µg/mL	*E. coli*	[42]
**Amphotericin B**	2 µg/mL	*A. niger*	[39]	**31j**	62.5 µg/mL	*E. coli*	[42]
**Fluconazole**	2 µg/mL	*A. niger* and *P. chrysogenum*	[39]	**31k**	62.5 µg/mL	*S. aureus*	[42]
**28a**	50 µg/mL	*C. albicans* and *A. niger*	[40]	**31l**	200 µg/mL	*B. subtilis*	[42]
**28b**	50 µg/mL	*C. albicans*	[40]	**Ampicillin**	100 µg/mL	*E. coli* and *S. typhi*	[42]
**28c**	25 µg/mL	*C. albicans* and *A. flavus*	[40]	**Chloramphenicol**	50 µg/mL	*B. subtilis*, *S. aureus*, *E. coli*, and *S. typhi*	[42]
**28d**	25 µg/mL	*C. albicans* and *F. oxysporum*	[40]	**Norfloxacin**	10 µg/mL	*S. aureus*, *E. coli*, and *S. typhi*	[42]
**28e**	12.5 µg/mL	*F. oxysporum*	[40]	**Griseofulvin**	100 µg/mL	*A. niger*	[42]
**28f**	12.5 µg/mL	*C. albicans*	[40]	**Nystatin**	100 µg/mL	*A. niger* and *C. albicans*	[42]
**28g**	50 µg/mL	*C. albicans* and *F. oxysporum*	[40]	**32a**	18 mm	*P. aeruginosa*	[43]
**28h**	25 µg/mL	*C. albicans*	[40]	**32b**	14 mm	*S. aureus* and *K. pneumoniae*	[43]
**Miconazole**	12.5 µg/mL	*A. flavus*	[40]	**32c**	15 mm	*S. aureus*	[43]
**Fluconazole**	6.25 µg/mL	*F. oxysporum* and *A. flavus*	[40]	**32e**	13 mm	*E. coli*	[43]
**29g**	128 µg/mL	*E. faecalis*	[41]	**32f**	13 mm	*K. pneumoniae*	[43]
**29i**	256 µg/mL	*E. faecalis*	[41]	**33a**	15 mm	*S. aureus*	[43]
**29l**	256 µg/mL	*E. faecalis*	[41]	**33e**	15 mm	*K. pneumoniae*	[43]
**29m**	64 µg/mL	*E. faecalis*	[41]	**33g**	17 mm	*E. coli*	[43]
**29n**	8 µg/mL	*E. faecalis*	[41]	**33h**	13 mm	*S. aureus*	[43]
**29o**	16 µg/mL	*E. faecalis*	[41]	**33j**	16 mm	*P. aeruginosa*	[43]
**29p**	64 µg/mL	*E. faecalis*	[41]	**33k**	15 mm	*E. coli*	[43]
**29q**	64 µg/mL	*E. faecalis*	[41]	**Pefloxacin**	36 mm	*S. aureus*	[43]
**29s**	64 µg/mL	*E. faecalis*	[41]				

In 2018, Savanur et al. [47] established new series of coumarin, quinolinone, and benzyl-linked 1,2,3-triazole derivatives (**38a**–**b**, **39a**–**k**, **40a**–**g**, **41a**–**f**) via click chemistry, as portrayed in Figure 6, and subjected the molecules to antimicrobial studies. Synthesized coumarin–triazole compounds were screened for antibacterial studies against Gram-positive bacteria, *E. coli* (NCIM 5346), *P. aeruginosa* (NCIM 5514), and *B. bronchiseptica* (NCIM 5346), and Gram-negative bacteria, *S. aureus* (NCIM 5345), *B. subtilis* (NCIM 2920), and (NCIM 5346) (Table 4). With a MIC of 1.0 µg/mL, compound **39j** with chloro and methoxy substitution on coumarin was extremely effective against *S. aureus* and *P. aeruginosa*. Additionally, compound **39j** exhibited excellent activity with MICs of 8.0 µg/mL, 16 µg/mL, and 16 µg/mL against *B. subtilis*, *B. cereus*, and *B. bronchiseptica*, respectively. Apart from compound **39j**, compounds **40g** (chloro substitution at C-6 on coumarin and 1-azacoumarin) and **41f** (chloro-substituted triazoles with benzyl group) demonstrated excellent activity against *S. aureus* with MICs of 1.0 µg/mL, which is comparable to the standard dose of ciprofloxacin (1.0 µg/mL). Further, the molecules tested for their antifungal assay against eight *Candida* fungal strain species (yeast specimens), included *C. albicans*, *C. tropicalis*, *C. utilis*, *C. krusei*, and *Aspergillus species* (*filamentous fungi*), such as *A. fumigatus*, *A. niger*, *R. oryzae*, and *R. bataticola*. Of all the compounds tested, **39i** and **39j** (with chloro and methoxy substitution) were highly active with MIC 1.0 µg/mL against *Candida* species. Compound **39e** was excellent with MICs of 1.0 µg/mL and MIC of 2.0 µg/mL against *C. krusei* and *C. albicans*, respectively. Furthermore, **40f**, a quinolinone analog with methyl substitution, was found to be a highly-active compound against *C. albicans*, *C. utilis*, and *C. krusei* with MICs 1.0 µg/mL, 2.0 µg/mL, and 4.0 µg/mL, respectively. Additionally, the same compound (**40f**) was also found to be very active against *A. niger* with MIC of 1.0 µg/mL. The in silico analysis showed that the active compounds (**39f** and **39h**) bind to the active sites of the two antifungal target proteins (1FI4 and 3LD6). Interestingly, compound **39h** showed the highest binding affinity (−11.0 kcal/mol) toward 1FI4, whereas **39f** displayed favorable interaction (−12.5 kcal/mol) toward 3LD6. The authors believe that these compounds represent a new platform for antimicrobial activity and could be further optimized therapeutically. 

**Table 4:** Antimicrobial activity data of reported coumarin triazole derivatives.

Compound	Activity Observed	Bacteria/Fungal	Ref.	Compound	Activity Observed	Bacteria/Fungal	Ref.
**35a**	25 (10) µg/mL	*B. subtilis* and *E. coli*	[45]	**42f**	12 µg/mL	*S. aureus*	[48]
**35b**	25 (13) µg/mL	*S. aureus* and *E. coli*	[45]	**42g**	11 µg/mL	*S. aureus*	[48]
**35c**	12.5 (12) µg/mL	*B. subtilis*	[45]	**42h**	9 µg/mL	*S. aureus*	[48]
**35d**	6.25 (15) µg/mL	*S. aureus*	[45]	**42i**	12 µg/mL	*E. coli*	[48]
**35e**	6.25(15) µg/mL	*B. subtilis*, *S. aureus*, and *P. vulgaris*	[45]	**42j**	7 µg/mL	*S. aureus*	[48]
**35f**	25(12) µg/mL	*S. aureus* and *E. coli*	[45]	**42k**	11 µg/mL	*S. aureus* and *E. coli*	[48]
**35g**	12.5 (12) µg/mL	*B. subtilis* and *S. aureus*	[45]	**42l**	18 µg/mL	*E. coli*	[48]
**35h**	6.25 (15) µg/mL	*S. aureus* and *E. coli*	[45]	**43a**	7.5 µg/mL	*E. coli* and *P. aeruginosa*	[49]
**35i**	6.25 (15) µg/mL	*B. subtilis*, *S. aureus*, and *E. coli*	[45]	**43b**	5.5 µg/mL	*E. coli*	[49]
**35j**	3.125 (19) µg/mL	*B. subtilis*, *S. aureus*, and *E. coli*	[45]	**43c**	6.5 µg/mL	*E. coli*	[49]
**Gentamicin**	1.56 (31) µg/mL	*B. subtilis*, *S. aureus*, and *E. coli*	[45]	**Ciprofloxacin**	4.5 µg/mL	*K. pneumoniae*	[49]
**Fluconazole**	3.125 (25) µg/mL	*A. niger* and *C. albicans*	[45]	**44a**	0.8 µg/mL	*M. tuberculosis*	[50]
**36a**	50 µg/mL	*E. faecalis*	[46]	**44b**	1.6 µg/mL	*M. tuberculosis*	[50]
**36b**	12.5 µg/mL	*E. faecalis*	[46]	**44c**	1.6 µg/mL	*M. tuberculosis*	[50]
**36c**	100 µg/mL	*E. faecalis*	[46]	**44d**	1.6 µg/mL	*M. tuberculosis*	[50]
**36d**	200 µg/mL	*E. faecalis*	[46]	**44e**	1.6 µg/mL	*M. tuberculosis*	[50]
**36e**	100 µg/mL	*E. faecalis*	[46]	**44f**	3.12 µg/mL	*M. tuberculosis*	[50]
**36f**	50 µg/mL	*E. faecalis*	[46]	**44g**	6.25 µg/mL	*M. tuberculosis*	[50]
**36g**	100 µg/mL	*E. faecalis*	[46]	**44h**	1.6 µg/mL	*M. tuberculosis*	[50]
**36h**	400 µg/mL	*S. aureus* and *E. faecalis*	[46]	**44i**	12.5 µg/mL	*M. tuberculosis*	[50]
**36i**	200 µg/mL	*E. faecalis*	[46]	**Pyrazinamide**	3.12 µg/mL	*M. tuberculosis*	[50]
**36j**	800 µg/mL	*S. aureus* and *E. faecalis*	[46]	***Streptomycin***	6.25 µg/mL	*M. tuberculosis*	[50]
**36k**	400 µg/mL	*E. faecalis*	[46]	**Ciprofloxacin**	3.12 µg/mL	*M. tuberculosis*	[50]
**36l**	400 µg/mL	*E. faecalis*	[46]	**45a**	2.5 ± 0.2 cm	*Penicillium* sp.	[51]
**37a**	400 µg/mL	*E. faecalis*	[46]	**45b**	2.5 ± 0.5 cm	*S. aureus*	[51]
**37b**	800 µg/mL	*E. faecalis* and *K. pneumoniae*	[46]	**45c**	2.1 ± 0.4 cm	*S. aureus*	[51]
**37c**	400 µg/mL	*E. faecalis*	[46]	**45d**	1.7 ± 0.6 cm	*S. aureus*	[51]
**37d**	100 µg/mL	*E. faecalis*	[46]	**45e**	1.8 ± 0.4 cm	*Penicillium* sp.	[51]
**37e**	100 µg/mL	*E. faecalis*	[46]	**45f**	1.4 ± 0.3 cm	*Penicillium* sp.	[51]
**37f**	200 µg/mL	*C. albicans*	[46]	**45g**	1.2 ± 0.6 cm	*Penicillium* sp.	[51]
**37g**	200 µg/mL	*S. aureus*	[46]	**46a**	1.7 ± 0.4 cm	*Penicillium* sp.	[51]
**37h**	50 µg/mL	*E. faecalis*	[46]	**46b**	1.3 ± 0.6 cm	*Penicillium* sp.	[51]
**37i**	100 µg/mL	*E. faecalis*	[46]	**46c**	1.5 ± 0.4 cm	*Penicillium* sp.	[51]
**37j**	800 µg/mL	*S. aureus* and *E. faecalis*	[46]	**46d**	1.0 ± 0.4 cm	*Penicillium* sp.	[51]
**37k**	50 µg/mL	*E. faecalis*	[46]	**46e**	1.1 ± 0.3 cm	*S. enterica*	[51]
**37l**	800 µg/mL	*E. faecalis*	[46]	**46f**	0.7 ± 0.1 cm	*S. enterica*	[51]
**Chloramphenicol**	1.2 µg/mL	*E. coli*	[46]	**46g**	0.5 ± 0.1 cm	*E. coli*	[51]
**Ketoconazole**	8 µg/mL	*C. albicans*	[46]	**47a**	1.1 ± 0.2 cm	*S. enterica*	[51]
**38a**	31.25 µg/mL	*S. aureus* and *B. subtilis*	[47]	**47b**	0.6 ± 0.1 cm	*S. aureus*	[51]
**38b**	16 µg/mL	*S. aureus*	[47]	**47c**	0.5 ± 0.2 cm	*S. aureus*	[51]
**39a**	16 µg/mL	*B. subtilis* and *B. cereus*	[47]	**47d**	1.1 ± 0.1 cm	*S. enterica*	[51]
**39b**	31.25 µg/mL	*B. subtilis*	[47]	**47e**	0.7 ± 0.2 cm	*F. oxysporum*	[51]
**39c**	8 µg/mL	*S. aureus*	[47]	**47f**	0.6 ± 0.1 cm	*M. smegmatis*	[51]
**39d**	8 µg/mL	*B. subtilis*	[47]	**47g**	0.5 ± 0.1 cm	*E. coli*	[51]
**39e**	4 µg/mL	*S. aureus*	[47]	**48a**	>1000 µg/mL	*S. aureus*	[52]
**39f**	31.25 µg/mL	*S. aureus*	[47]	**48b**	416.7 ± 60.09 µg/mL	*S. aureus*	[52]
**39g**	8 µg/mL	*S. aureus* and *B. subtilis*	[47]	**48c**	0.16 ± 0.08 µg/mL	*S. aureus*	[52]
**39h**	4 µg/mL	*S. aureus*	[47]	**Ceftriaxonum**	0.97 ± 0.02 µg/mL	*S. aureus*	[52]
**39i**	8 µg/mL	*S. aureus*	[47]	***Streptomycin***	1.89 ± 0.08 µg/mL	*S. aureus*	[52]
**39j**	1 µg/mL	*S. aureus* and *P. aeruginosa*	[47]	**62a**	250 ± 20.41 µg/mL	*S. aureus*	[52]
**39k**	16 µg/mL	*S. aureus*	[47]	**62b**	425 ± 47.87 µg/mL	*S. aureus*	[52]
**40a**	16 µg/mL	*P. aeruginosa*	[47]	**62c**	51.25 ± 3.15 µg/mL	*S. aureus*	[52]
**40b**	16 µg/mL	*P. aeruginosa*	[47]	**63a**	>1000 µg/mL	*S. aureus*	[52]
**40c**	16 µg/mL	*S. aureus*	[47]	**63b**	>1000 µg/mL	*S. aureus*	[52]
**40d**	8 µg/mL	*S. aureus* and *B. subtilis*	[47]	**63c**	0.31 ± 0.23 µg/mL	*S. aureus*	[52]
**40e**	8 µg/mL	*S. aureus* and *P. aeruginosa*	[47]	**64a**	0.03 µg/mL	*C. albicans*	[53]
**40f**	4 µg/mL	*S. aureus* and *P. aeruginosa*	[47]	**64b**	0.015 µg/mL	*C. albicans* and *C. parapsilosis*	[53]

Kolichala et al. [48] reported the regioselective synthesis and antibacterial activity of 6-[(l-ethyl-l*H*-l,2,3-triazol-4-yl)methoxy]-4-methyl-2*H*-chromen-2-ones (**42a**–**l**), as depicted in Figure 6 (Table 4). The disclosed compounds were examined using the paper disc technique against the bacterial strains *E. coli* (Gram-negative) and *S. aureus* (Gram-positive). According to the authors, each analog exhibited good to moderate activity. The compounds **42b**, **42e**, **42f**, **42g**, **42i**, **42h**, and **42l** among the studied compounds showed relatively moderate to exceptional activity (MIC range 8–32 µg/mL), but they did not compare standard drugs in this study. Chityala et al. [49] reported the synthesis and antibacterial activity of coumarin-1,2,3-triazoles (**43a**–**c**) (Figure 6) (Table 4). The compounds were evaluated for antibacterial assay against bacterial strains *E. coli*, *K. pneumonia*, *P. aeruginosa*, *S. aureus*, and *S. pyogenes*. Compounds **43a**–**c** portrayed excellent results, as confirmed by their MIC values ranging from 5.5–17.5µg/mL. PEG-400 was used as an environmentally acceptable catalyst by Shaikh et al. [50] to explain the synthesis and antibacterial activity of a series of substituted coumarin-1,2,4-triazolidine-3-thiones **44a**–**i** (Figure 6). Gram-positive (*S. aureus*, *B. subtilis*), Gram-negative (*E. coli*, *P. aeruginosa*), and four fungus strains (*C. albicans*, *A. niger*, *A. flavus*, and *A. fumigatus*) were used to assess the antibacterial activity of all the adducts. Excellent antibacterial activity was revealed by compounds **44a**, **44b**, **44c**, **44h**, **44i**, **44a**, and **44b** against *S. aureus*, *B. subtilis*, and *E. coli* strains with MICs ranging from 0.8 to 1.6 µg/mL. All the tested substances had a mediocre effect on the *P. aeruginosa* bacterial strain. To elucidate the interaction mechanism of these compounds with target proteins, authors performed molecular docking studies and identified the target protein of *E. coli* FabH (Fatty acid biosynthesis, enzyme H). The compound **44d** docked well, and three important hydrogen bonding interactions were shown (PDB ID 1HNJ) in this study. 

Bhagat et al. [51] synthesized a library of indolinedione–coumarin hybrids **45a**–**g**, **46a**–**g,** and **47a**–**g** (Figure 6) (Table 4). All the synthesized hybrid molecules were screened for antibacterial assay against two Gram-positive bacteria (*S. aureus*, *M. smegmatis*) and two Gram-negative bacteria (*E. coli*, *S. enteric*). Among these tested microorganisms, *S. aureus* was the most sensitive, and *E. coli* was the most resistant one. Among all the compounds (**45a**–**g**) tested, **45b** arose as the most potent one with ZoI of 2.5 and 1.3 cm for bacterial strains, *S. aureus* and *S. enteric*, respectively. Additionally, compounds **45a**–**g** were tested for antifungal studies against four fungal strains (*C. albicans*, *A. mali*, *Penicillium* sp., and *F. oxysporum*). Of all the molecules, **45a** (ZoI 2.5 cm) and **45b** (ZoI 1.3 cm) exhibited excellent antifungal activity for the fungal strain *Penicillium* sp. The molecular docking studies revealed the probable mechanism of action of these analogs. The docking studies displayed binding interactions of **45b** within the catalytic active site of *S. aureus* DHFR. This potent indolinedione–coumarin hybrid **45b** could be further developed as an antimicrobial agent.

**Figure 7:**
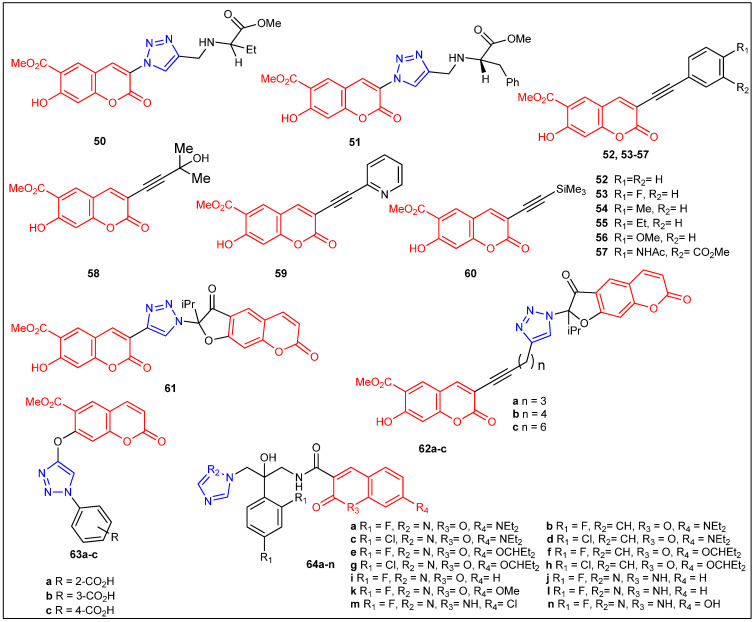
Structures of the reported coumarin triazole derivatives from **2018**–**2019**.

In 2019 Lipeeva et al. [52] reported the synthesis of 1,2,3-triazoles-linked coumarin and 1,2,3-triazolyl or 1,2,3-triazolylalk-1-inyl-linked coumarin–2,3-furocoumarin hybrids (**48a**–**c**, **49**–**61**, **62a**–**c**, and **63a**–**c**) (Figure 6 and Figure 7) and evaluated for their in vitro antibacterial activity against the strains *S. aureus*, *B. subtilis*, *A. viscosus*, and *E. coli*. Coumarin-benzoic acid hybrids **48c** (MIC 0.16 µg/mL), **63c** (MIC 0.31 µg/mL), and compound **57**, **non-triazole-coumarin analog** (MIC 0.41 µg/mL), showed promising inhibition against *S. aureus*. Furthermore, 1,2,3-triazolyloct-1-inyl-linked coumarin–2,3-furocoumarin hybrid **62c** (MIC 0.02 μg/mL) demonstrated excellent activity toward *B. subtilis*. In the same year, Elias et al. reported coumarin and quinoline-based antifungal azole derivatives (**64a**–**n**), as depicted in Figure 7 (Table 4). All molecules were screened against a series of *Candida* pathogens: *C. albicans* 90028, *C. albicans* P-87, *C. albicans* SN152, *C. glabrata* 66032, *C. glabrata* 2001, *C. glabrata* 192, *C. parapsilosis* 90018, *C. parapsilosis* 22019, *C. guilliermondii T-47*, *C. dubliniensis T-99*. The newly prepared imidazole or triazole-bearing coumarins have shown MIC 0.03 to 63 μg/mL toward tested fungal strains. The biological findings revealed that imidazole-bearing antifungals were more efficient than analogs derived from triazoles in reducing the lagging proliferation linked to the retention and/or recurrence of fungal infections [53].

**Figure 8:**
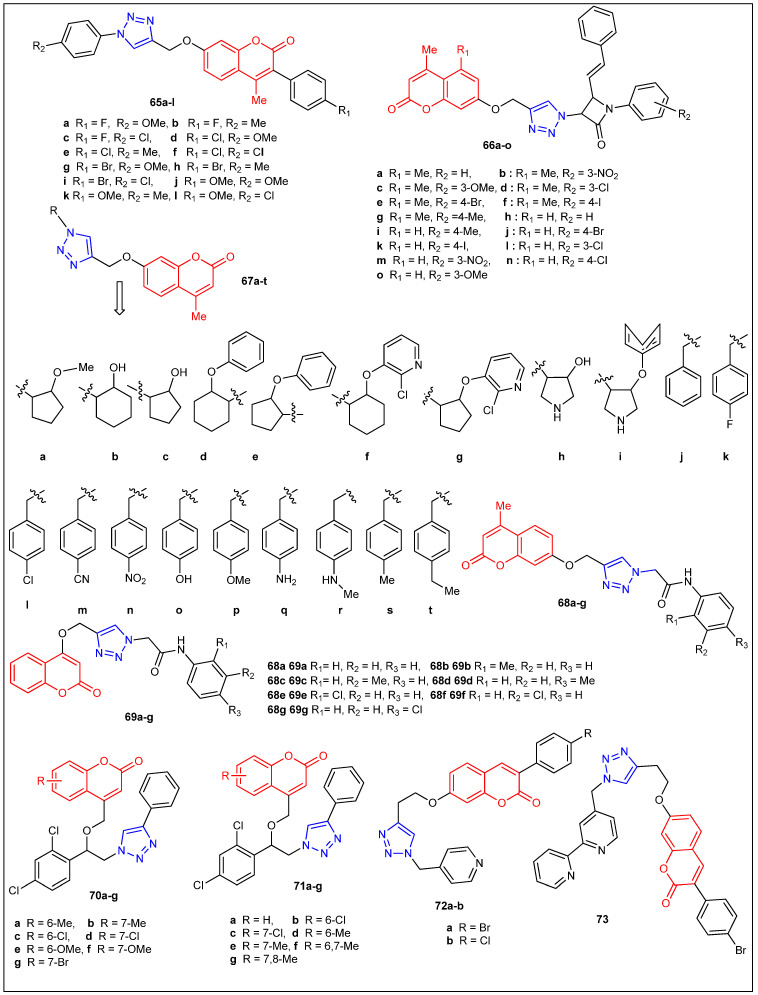
Structures of the reported coumarin triazole derivatives from **2020**–**2021**.

From copper(I)-catalyzed click reaction between various substituted terminal alkynes and arylazides, coumarin-based 1,4-disubstituted 1,2,3-triazoles [**65a**–**l**] (Figure 8) were synthesized through microwave irradiation [54]. All the prepared compounds were screened for their antibacterial potential against *S. aureus*, *E. coli*, *B. subtilis*, and *K. pneumonia* at concentrations of 10 µg mL^−1^ and 20 µg mL^−1^, respectively. Amongst all the newly prepared coumarin triazoles, **65a** (32 mm), **65d** (32 mm), **65g** (34 mm), and **65j** (34 mm) were highly active toward *E. coli* because of the presence of the methoxy group in the triazole ring. Furthermore, compounds **65k** (26 mm) and **65l** (27 mm) have demonstrated nearly similar activity to that of the standard drug gatifloxacin (30 mm). Synthesized compounds [**65a**–**l**] were also screened for their in vitro antifungal potential through three fungal organisms such as *A. flavus*, *F. sporum*, and *A. niger*, at a concentration of 50 µg mL^−1^, and the results with ZoI range from 10.3mm to 18.8mm and have been mostly comparable to the standard drug Clotrimazole (Table 5). It was noticed that among all the prepared compounds, **65a**, **65b**, **65c**, **65j**, **65k**, and **65l** exhibited good activity through three pathogenic fungi due to the presence of fluorine and methoxy groups on coumarin and triazole rings. The remaining compounds displayed comparable activity to Clotrimazole as a standard drug. In this series of compounds, the chloro and bromo halogens, along with the methoxy substitutions on both phenyl rings, seem to be important for obtaining comparable antimicrobial activity. Singh et al. reported the synthesis and antimicrobial evaluation of a series of new coumarin-tagged *β*-lactam triazole hybrids [**66a**–**o**] [55] (Figure 8). Antimicrobial activity studies concluded that compounds containing chloro and methyl groups (**66c** and **66i**) exhibited moderate antimicrobial activity toward *P. aeruginosa* (18.97% inhibition at 32 µg/mL) and *C. albicans* (21.65% inhibition at 32 µg/mL) strains, respectively. Conversely, all the screened compounds were found to be less active than the standard drugs, such as Colistin and Vancomycin for bacterial and Fluconazole for fungal strains (Table 5).

**Table 5:** Antimicrobial activity data of reported coumarin triazole derivatives.

Compound	Activity Observed	Bacteria/Fungal	Ref.	Compound	Activity Observed	Bacteria/Fungal	Ref.
**65a**	23 mm	*B. subtilis*	[54]	**67i**	50 μg/mL	*P. aeruginosa*	[56]
**65b**	16 mm	*B. subtilis*	[54]	**67k**	5 μg/mL	*S. aureus*	[56]
**65c**	18 mm	*S. aureus*	[54]	**67l**	25 μg/mL	*P. aeruginosa*	[56]
**65d**	23 mm	*S. aureus*	[54]	**67m**	10 μg/mL	*P. aeruginosa*	[56]
**65e**	16 mm	*S. aureus*	[54]	**67p**	50 μg/mL	*B. subtilis*	[56]
**65f**	19 mm	*S. aureus*	[54]	**67s**	50 μg/mL	*B. subtilis*	[56]
**65g**	24 mm	*S. aureus*	[54]	**67t**	75 μg/mL	*P. aeruginosa*	[56]
**65h**	16 mm	*B. subtilis*	[54]	**Ciprofloxacin**	0.2 μg/mL	*S. aureus*	[56]
**65i**	19 mm	*B. subtilis*	[54]	**Fluconazole**	10 μg/mL	*A. flavus*	[56]
**65j**	27 mm	*B. subtilis*	[54]	**68a**	12.5 μg/mL	*A. niger*	[57]
**65k**	19 mm	*S. aureus*	[54]	**68b**	12.5 μg/mL	*A. niger* and *C. neoformans*	[57]
**65l**	19 mm	*S. aureus*	[54]	**68c**	12.5 μg/mL	*C. albicans*	[57]
**Gatifloxacin**	20 mm	*S. aureus* and *B. subtilis*	[54]	**68d**	12.5 μg/mL	*A. flavus* and *A. niger*	[57]
**66b**	10.44 mm	*P. aeruginosa*	[55]	**68e**	12.5 μg/mL	*C. albicans* and *A. niger*	[57]
**66c**	18.97 mm	*P. aeruginosa*	[55]	**68f**	25 μg/mL	*A. niger* and *C. neoformans*	[57]
**66d**	14.96 mm	*C. albicans*	[55]	**68g**	25 μg/mL	*C. albicans* and *F. oxysporum*	[57]
**66e**	4.35 mm	*C. albicans*	[55]	**69a**	25 μg/mL	*F. oxysporum*, *A. flavus*, and *C. neoformans*	[57]
**66f**	17.78 mm	*P. aeruginosa*	[55]	**69b**	12.5 μg/mL	*C. albicans*, *A. flavus*, *A. niger*, and *C. neoformans*	[57]
**66g**	11.11 mm	*P. aeruginosa*	[55]	**69c**	12.5 μg/mL	*F. oxysporum* and *A. niger*	[57]
**66h**	12.11 mm	*P. aeruginosa*	[55]	**69d**	12.5 μg/mL	*A. flavus*	[57]
**66i**	21.65 mm	*C. albicans*	[55]	**69e**	12.5 μg/mL	*C. albicans*, *F. oxysporum*, *A. flavus*, and *A. niger*	[57]
**66j**	9.42 mm	*C. albicans*	[55]	**69f**	12.5 μg/mL	*F. oxysporum*, *A. flavus*, and *A. niger*	[57]
**66k**	7.32 mm	*P. aeruginosa*	[55]	**69g**	12.5 μg/mL	*C. neoformans*	[57]
**66l**	16.37 mm	*P. aeruginosa*	[55]	**70a**	16 μg/mL	*S. aureus*	[58]
**66m**	7.74 mm	*P. aeruginosa*	[55]	**70b**	31.25 μg/mL	*S. aureus* and *E. coli*	[58]
**66n**	6.66 mm	*P. aeruginosa*	[55]	**70c**	4 μg/mL	*S. aureus*	[58]
**66o**	8.47 mm	*P. aeruginosa*	[55]	**70d**	4 μg/mL	*S. aureus*	[58]
**67a**	50 μg/mL	*B. subtilis*	[56]	**70e**	8 μg/mL	*S. aureus* and *P. aeruginosa*	[58]
**67f**	10 μg/mL	*E. coli*, *S. aureus*, and *P. aeruginosa*	[56]	**70f**	16 μg/mL	*S. aureus*	[58]
**67g**	10 μg/mL	*E. coli*, *S. aureus*, *P. aeruginosa* and *B. subtilis*	[56]	**70g**	16 μg/mL	*S. aureus*	[58]

Joy et al. synthesized coumarins linked with 1,2,3-triazoles [**67a**–**t**] (Figure 8) under microwave irradiation and evaluated their antimicrobial activity (Table 5) [56]. The coumarins linked with 1,2,3-triazoles (**67k**) (5 μg/mL MIC) and (**67g**) (10 μg/mL MIC) revealed good antibacterial activity compared with the standard drug Ciprofloxacin (0.2 μg/mL MIC) against all the tested bacteria. Additionally, **67n** (150 μg/mL MIC) displayed better antifungal activity compared to other prepared coumarins linked with 1,2,3-triazoles but was not promising when compared with the standard drug fluconazole (20 μg/mL MIC). A series of new 1,2,3-triazole-tethered coumarin conjugates [**68a**–**g** and **69a**–**g**] (Figure 8) (Table 5) were prepared via the click chemistry approach in excellent yields and screened for their antifungal activity toward five fungal strains such as *C. albicans*, *F. oxysporum*, *A. flavus*, *A. niger* and *C. neoformans* [57]. Furthermore, 1,2,3-triazole-tethered coumarin conjugates **68b**, **68d**, **68e**, **69b**, and **69e** demonstrated excellent antifungal activity with MIC values ranging from 12.5 to 25 μg/mL compared with the standard drug miconazole with lower MIC values. The molecular docking studies of novel triazole–coumarin conjugates disclosed that they have a high affinity toward the active site of enzyme P450 cytochrome lanosterol 14α-demethylase. This docking study offers a new platform for the structure-based drug design development for antimicrobial agents. Kalkhambkar et al. reported the antimicrobial activity of coumarin- and 1-azacoumarin-linked triazoles against four bacterial and six fungal microorganisms [58]. Among them, chloro-substituted coumarin (**70c**) (4 μg/mL MIC) and azacoumarin (**70b**) (16 μg/mL MIC) compounds exhibited the highest antibacterial activity toward *S. aureus*. On the other hand, methyl (**71b**) (4 μg/mL MIC) and bromo-substituted coumarin (**70g**) (6 μg/mL MIC) demonstrated better antifungal activity against *C. utills* and *C. krusei*, whereas dimethyl-substituted azacoumarins (**70f** and **71g**) (1.0 μg/mL MIC) exhibited comparable antifungal activity toward *C. albicans* compared to standard drugs Itraconazole and Miconazole. The design and synthesis of three new 3-arylcoumarin derivatives (**72a-b** and **73**) (Figure 8) were reported by Pavic et al. [59]. In addition, antibacterial activity studies were done against Gram-positive bacteria, three *S. aureus* strains, including methicillin-resistant *S. aureus* (MRSA), *E. faecium*, and *L. monocytogenes*, Gram-negative bacterial strain *P. aeruginosa*, and four *Candida* species including *C. albicans*, *C. glabrata*, *C. krusei* and *C. parapsilosis*. Unfortunately, all three new 3-arylcoumarin derivatives (**72a**,**b**, and **73**) are virtually inactive against the pathogens.

**Figure 9:**
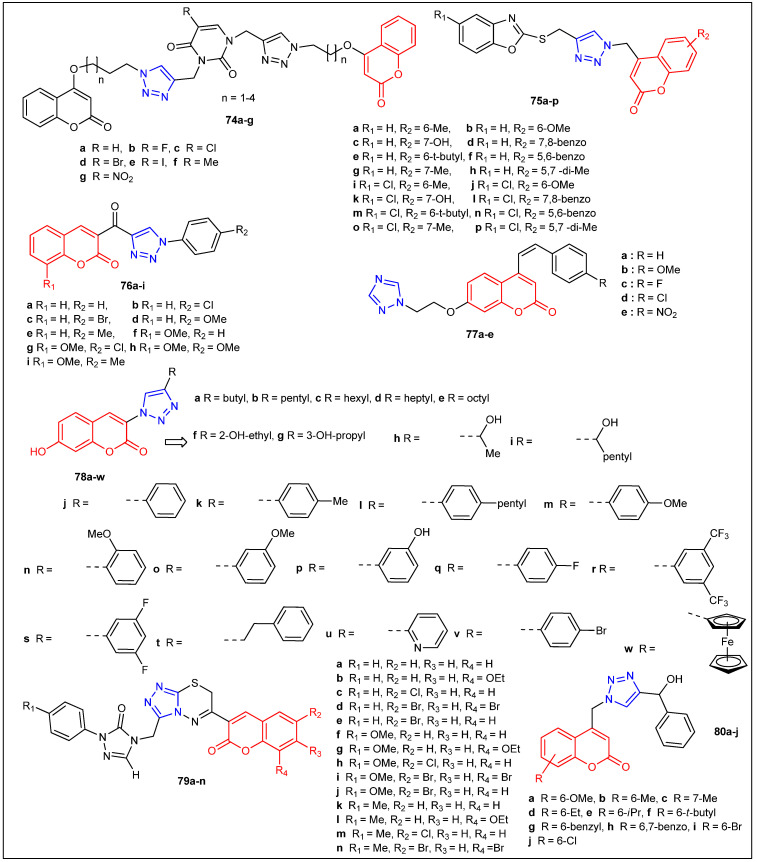
Structures of the reported coumarin triazole derivatives from **2021**–**2022**.

Uracil–coumarin hybrids (**74a**–**g**) (Figure 9) were screened for their antibacterial activities against a panel of drug-susceptible and drug-resistant Gram-negative and Gram-positive pathogens (Table 6). Antibacterial activities resulted in two lead molecules, **74b**, the fluoro substitution on a pyrimidine-dione ring (MIC = 11.7 μg/mL) and **74c**, the chloro substitution on a pyrimidine-dione ring (MIC = 7.23 μg/mL), which were found comparable to that of standard drug Levofloxacin’s MIC value of 3.12 μg/mL [60]. A series of new benzoxazole–coumarin-linked 1,2,3-triazoles (**75a**–**p**) (Figure 9) (Table 6) were prepared from conventional as well as microwave irradiation methods in good purity and yields and were studied for their antibacterial activity toward panel of Gram-positive and Gram-negative bacteria [61]. The benzoxazole–coumarin-linked 1,2,3-triazoles **75m** and **75o** displayed excellent antimicrobial results for all tested microorganisms at MICs ranging from 3.12 to 6.25 μg/mL in comparison with the marketed drugs. The antimicrobial activity results demonstrated that the compounds **75m** and **75o** highlighted the importance of the presence as well as the position of the methyl group. The antimicrobial activity of coumarin-tethered 1,2,3-triazoles (**76a**–**i**) was evaluated toward a panel of pathogenic microorganisms, including the bacterial pathogens *E. coli*, *B. subtilis*, *S. aureus*, and fungal stains *A. niger*, *A. flavus* and *C. albicans* by Kariyappa et al. Antimicrobial results indicate that the prepared coumarin-tethered 1,2,3-triazoles (**76a**–**i**) (Figure 9) showed medium to good antimicrobial activities with MIC values of 6.5–75.0 μg/mL toward bacteria and 12.5–100.0 μg/mL against fungal species. The results, which were comparable with the standard drugs, employed ciprofloxacin (12.5–25.0 μg/mL) against bacteria and nystatin (25.0–50.0 μg/mL) toward fungi [62]. Narkhede et al. reported the preparation and antimicrobial activity of coumarin triazole derivatives (**77a**–**e**) (Figure 9) (Table 6). All coumarin triazole derivatives (**77a**–**e**) displayed around 44–51% inhibition against *E. coli* and *S. aureus*, whereas they did not show any activity toward *S. typhi.* It should be noted that antifungal data revealed that compounds **77c** and **77d** established the broadest spectrum of inhibitory activity (74.07% and 66.66%) toward *A. flavus*. The remaining coumarin triazole derivatives **77c**, **77d**, and **77e** are inactive against *C. albicans*; **77a** and **77b** were inactive against *A. flavus* [63].

Synthesis of new hybrids of 3-(1,2,3)-trazolyl-coumarin derivatives [**78a**–**w**] (Figure 9) was reported by Kraljevic et al. [64]. All hybrids of 3-(1,2,3)-trazolyl-coumarin derivatives [**78a**–**w**] were tested toward Gram-positive *S. aureus*, *S. aureus* MRSA, *E. faecali*, *E. faecium* VRE, and Gram-negative *E. coli*, *K. pneumoniae*, *K. pneumoniae* ESBL, *P.* and *A. baumannii*. Undesirably, all 3-(1,2,3)-trazolyl-coumarin derivatives [**78a**–**w**] had MICs higher than 128 μg/mL against all tested bacterial species (Table 6). In 2022, Kamble et al. reported the synthesis of a series of new triazolothiadiazine–coumarin hybrid derivatives (**79a**–**n**) (Figure 9) through a green and versatile synthetic route using agro waste extract WELPSA catalyzed cyclocondensation [65]. All the synthesized compounds were screened in vitro for their antifungal activity against three pathogenic fungi strains viz., *A. niger*, *C. albicans*, and *P. citranum*. New triazolothiadiazine–coumarin hybrid derivatives 7**9a** (14 mm), 7**9d** (12 mm), 7**9f** (16 mm), 7**9j** (15 mm), and 7**9m** (11 mm) are good inhibitors for *A. niger*, whereas 7**9a** (16 mm), 7**9g** (14 mm), and 7**9m** (14 mm) are respective inhibitors for *C. albicans*, and compounds 7**9b** (10 mm), 7**9d** (12m m), and 7**9e** (11 mm) are decent inhibitors for *P. citranum* (Table 6). The remaining compounds have displayed hopeful results suggesting that triazolothiadiazine–coumarin hybrid analogs could be further developed as promising drug Candidates. 

**Table 6:** Antimicrobial activity data of reported coumarin triazole derivatives.

Compound	Activity Observed	Bacteria/Fungal	Ref.	Compound	Activity Observed	Bacteria/Fungal	Ref.
**74a**	10 ± 0.3 mm	*S. aureus*	[60]	**79b**	60 μg/mL	*P. citranum*	[65]
**74b**	26 ± 0.9 mm	*S. aureus*	[60]	**79c**	60 μg/mL	*A. niger*	[65]
**74c**	28 ± 1.2 mm	*S. aureus*	[60]	**79d**	40 μg/mL	*A. niger* and *P. citranum*	[65]
**74d**	24 ± 1.1 mm	*S. aureus*	[60]	**79e**	60 μg/mL	*P. citranum*	[65]
**74e**	25 ± 1.0 mm	*S. aureus*	[60]	**79f**	40 μg/mL	*A. niger*	[65]
**74f**	16 ± 0.7 mm	*S. aureus*	[60]	**79g**	60 μg/mL	*C. albicans*	[65]
**74g**	20 ± 0.9 mm	*S. aureus*	[60]	**79h**	80 μg/mL	*C. albicans*	[65]
**76a**	25.0 ± 0.50 μg/mL	*E. coli*	[62]	**79i**	60 μg/mL	*A. niger* and *P. citranum*	[65]
**76b**	12.5 ± 0.45 μg/mL	*S. aureus*	[62]	**79j**	40 μg/mL	*A. niger*	[65]
**76c**	>100.0 μg/mL	*S. aureus*, *E. coli*, *P. aeruginosa*, and *C. albicans*	[62]	**79k**	60 μg/mL	*P. citranum*	[65]
**76d**	37.5 ± 0.80 μg/mL	*S. aureus*	[62]	**79l**	60 μg/mL	*P. citranum*	[65]
**76e**	25.0 ± 0.85 μg/mL	*P. aeruginosa*	[62]	**79m**	40 μg/mL	*A. niger*	[65]
**76f**	37.5 ± 1.60 μg/mL	*E. coli*	[62]	**79n**	60 μg/mL	*A. niger*	[65]
**76g**	6.5 ± 0.40 μg/mL	*P. aeruginosa*	[62]	**Fluconazole**	40 μg/mL	*A. niger*, *C. albicans*, and *P. citranum*	[65]
**76h**	>100.0 μg/mL	*S. aureus*, *E. coli*, *P. aeruginosa*, and *C. albicans*	[62]	**80a**	18.75 μg/mL	*B. subtilis*	[66]
**76i**	>100.0 μg/mL	*S. aureus*, *E. coli*, and *P. aeruginosa*	[62]	**80b**	18.75 μg/mL	*S. aureus*	[66]
**Ciprofloxacin**	12.5 ± 0.35 μg/mL	*P. aeruginosa*		**80c**	>75 μg/mL	*S. aureus*, *B. subtilis*, and *K. pneumonia*	[66]
**Nystatin**	25.0 ± 0.45	*C. albicans*		**80d**	>75 μg/mL	*S. aureus*, *B. subtilis*, and *K. pneumonia*	[66]
**77a**	44.00 (11) mm	*C. albicans*	[63]	**80e**	>75 μg/mL	*S. aureus*	[66]
**77b**	32.00 (08) mm	*C. albicans*	[63]	**80f**	9.3 μg/mL	*S. aureus*, *B. subtilis*, and *E. coli*	[66]
**77c**	44.00 (11) mm	*P. aeruginosa*	[63]	**80g**	9.3 μg/mL	*B. subtilis* and *M. luteus*	[66]
**77d**	38.46 (10) mm	*E. coli*	[63]	**80h**	9.3 μg/mL	*B. subtilis* and *M. luteus*	[66]
**77e**	44.44 (12) mm	*S. aureus* and *A. flavus*	[63]	**80i**	>75 μg/mL	*B. subtilis*	[66]
**Strepto-mycin**	100 (25) mm	*P. aeruginosa* and *S. typhi*	[63]	**80j**	>75 μg/mL	*B. subtilis*	[66]
**Greseo-fulvin**	100 (25) mm	*C. albicans*	[63]	**Ampicillin**	4.6 μg/mL	*S. aureus*, *B. subtilis*, *M. luteus*, and *K. pneumonia*	[66]
**79a**	40 μg/mL	*A. niger*	[65]				

In the same year, synthesis and antimicrobial activity of a novel class of 4-[(40-hydroxymethylphenyl)-1H-10,20,30-triazol-1-yl-methyl]-2H-chromen-2-ones (**80a**–**j**) (Figure 9) were reported from Suresh et al. [66]. The investigation of the antimicrobial activities of the prepared coumarinyl-derivatives (**80a**–**j**) toward three Gram-positive bacterial strains, *S. aureus*, *B. subtilis*, *M. luteus*, and three Gram-negative bacterial strains, *E. coli*, *K. pneumonia*, *P. aeruginosa*, were carried out (Table 6). Few of the coumarin derivatives exhibited medium to good activity with MIC values ranging from 9.3–37.50 μg/mL in DMSO. However, compounds **80f** (9.3 mm, 9.3 mm, 18.75 mm, and 9.3), **80g** (18.75 mm, 9.3 mm, 9.3 mm, and 18.75 mm)**,** and **80h** (18.75 mm, 9.3 mm, 9.3 mm, and 18.75) displayed great activity against *S. aureus*, *B. subtilis*, *M. luteus*, and *E. coli*, respectively. This could be due to the existence of the *t*-butyl group/aromatic rings in the compounds **80f**, **80g**, and **80h**. The prepared compounds (**80a**–**j**) were also subjected to antifungal activity to determine their zone of inhibition. The antifungal activities have been completed with *A. fumigatus*, *T. vivide*, *C. lipolytic*, and *A. niger*. The coumarinyl derivatives **80f** (18 mm, and 18 mm), **80g** (20 mm, and 19 mm), and **80h** (20 mm, and 18 mm) are highly active toward the fungal strains *A. fumigatus* and *T. vivide*, respectively. However, medium activity was observed toward the other strains, *C. lipolytica* and *A. niger*. The antifungal potential trends are as follows: **80g** ≈ **80h** > **80f** > **80c** > **80b** > **80a** ≈ **80i** ≈ **80j** > **80d** > **80e**. In summary, antifungal properties follow the same pattern as discussed for the antibacterial properties [66]. The molecular docking studies using the most potent compounds **80f**, **80g**, and **80h** with N-terminal domain of DNA binding protein of *S. aureus* (4PQL), a long-chain secondary alcohol dehydrogenase protein of *M. luteus* (6QKN), and lipase of *B. subtilis* (1ISP) revealed their mechanism of action and produced improved activity. High binding affinity with target proteins confirms that these analogs are extremely active antibacterial agents.

## 3. Conclusions

The MDR strains are posing serious health threats, especially in developing countries. Therefore, there is a great need to develop novel antibiotics to overcome MDR microbial strains. The coumarin- and triazole-based compounds are potential structural motifs because of their drug-like properties and high therapeutic indexes. Both pharmacophores have been extensively utilized in the development of several clinical drugs. Medicinal chemists are now actively engaged in combining both coumarin and triazole moieties to obtain novel and highly effective single-molecule antibiotic drug Candidates. Our review abridges the known reports of various coumarin triazoles or triazole–coumarin derivatives and their antimicrobial activities. As summarized in the above sections, the presence of both coumarin and triazole functionalities in a single molecule has enhanced the efficacy of antimicrobial activities. The above information aims to aid the medical research community in developing novel, potent, and safe antimicrobial drug Candidates to combat the MDR in microbial diseases.

## Data Availability

The data are contained within the article.
